# Using polygenic scores in combination with symptom rating scales to identify attention-deficit/hyperactivity disorder

**DOI:** 10.1186/s12888-024-05925-7

**Published:** 2024-06-27

**Authors:** André Høberg, Berit Skretting Solberg, Tor-Arne Hegvik, Jan Haavik

**Affiliations:** 1 Department of Biomedicine, University of Bergen, Bergen, 5009 Norway; 2 Child- and adolescent psychiatric outpatient unit, Hospital Betanien, Bergen, Norway; 3 Clinic of Surgery, St. Olavs Hospital, Trondheim, Norway; 4 Department of Global Public Health and Primary Care, University of Bergen, Bergen, Norway; 5 Bergen Center for Brain Plasticity, Division of Psychiatry, Haukeland University Hospital, Bergen, Norway

**Keywords:** Polygenic score, Attention deficit hyperactivity disorder, Diagnosis, Prediction, Polygenic risk score, Genetics

## Abstract

**Background:**

The inclusion of biomarkers could improve diagnostic accuracy of attention-deficit/hyperactivity disorder (ADHD). One potential biomarker is the ADHD polygenic score (PGS), a measure of genetic liability for ADHD. This study aimed to investigate if the ADHD PGS can provide additional information alongside ADHD rating scales and examination of family history of ADHD to distinguish between ADHD cases and controls.

**Methods:**

Polygenic scores were calculated for 576 adults with ADHD and 530 ethnically matched controls. ADHD PGS was used alongside scores from the Wender-Utah Rating Scale (WURS) and the Adult ADHD Self-Report Scale (ASRS) as predictors of ADHD diagnosis in a set of nested logistic regression models. These models were compared by likelihood ratio (LR) tests, Akaike information criterion corrected for small samples (AICc), and Lee R². These analyses were repeated with family history of ADHD as a covariate in all models.

**Results:**

The ADHD PGS increased the variance explained of the ASRS by 0.58% points (pp) (R^2^_ASRS_ = 61.11%, R^2^_ASRS + PGS_=61.69%), the WURS by 0.61pp (R^2^_WURS_ = 77.33%, R^2^_WURS + PGS_= 77.94%), of ASRS and WURS together by 0.57pp (R^2^_ASRS + WURS_=80.84%, R^2^_ASRS + WURS+PGS_=81.40%), and of self-reported family history by 1.40pp (R^2^_family_ = 28.06%, R^2^_family + PGS_=29.46%). These increases were statistically significant, as measured by LR tests and AICc.

**Conclusion:**

We found that the ADHD PGS contributed additional information to common diagnostic aids. However, the increase in variance explained was small, suggesting that the ADHD PGS is currently not a clinically useful diagnostic aid. Future studies should examine the utility of ADHD PGS in ADHD prediction alongside non-genetic risk factors, and the diagnostic utility of the ADHD PGS should be evaluated as more genetic data is accumulated and computational tools are further refined.

**Supplementary Information:**

The online version contains supplementary material available at 10.1186/s12888-024-05925-7.

## Introduction

A clinical diagnosis of attention-deficit/hyperactivity disorder (ADHD) relies on a detailed assessment of symptoms and patient history by a trained professional [1]. Structured interviews and rating scales of ADHD symptoms are effective aids in the diagnosis of ADHD and are commonly employed in both primary care and specialist treatment settings [1, 2]. Rating scales are cost-effective, provide standardized information on symptom severity, and allow for ADHD symptoms to be followed up to monitor clinical course and treatment efficacy [3].

ADHD has a high heritability, and genome-wide association studies (GWASs) have identified common genetic markers associated with the condition [4, 5]. A polygenic score (PGS) is a measure of an individual’s genetic liability for a trait [6]. It is calculated by summing up an individual’s common risk alleles weighed by their effect sizes given by a GWAS [6]. The ADHD PGS has several potential clinical applications, such as in screening, risk stratification, treatment optimization, and disease prediction [7–10]. The inclusion of the ADHD PGS in the diagnostic process could make the diagnosis of ADHD more precise.

This study aimed to investigate whether the ADHD PGS has potential as a diagnostic aid in ADHD diagnostics by providing additional information to self-report rating scales commonly used in clinical practice, as well as self-reported ADHD family history.

## Method

### Participants

Participants were recruited as part of the “ADHD in Norwegian Adults” project launched in 2004, and all participants provided a signed informed consent [11]. Data collection was performed with questionnaires mailed to participants between 2005 and 2008.

The case sample consists of a well-characterized group of patients, mainly recruited from a national registry of adults diagnosed with ADHD in Norway between 1997 and 2005. Inclusion criteria were a clinical diagnosis of ADHD and age greater than 18 years, and there were no formal exclusion criteria.

The controls were recruited from the general adult Norwegian population and were recruited from several sources. The majority (79%) were randomly selected individuals between 18 and 40 years of age from the Medical Birth Registry of Norway, which includes all individuals born in Norway after January 1st 1967 [12]. The remainder were recruited from friends of cases and from students attending the University of Bergen between 2005 and 2007. The inclusion criterium was age older than 18 years, and there were no exclusion criteria. For a more detailed description of the sample, see Johansson et al. 2008 [11].

### Genetic data

DNA was collected via kits for collecting saliva mailed to the participants. DNA from saliva samples were genotyped in two batches: one at the Broad Institute (Boston, MA, USA), genotyped on the Human OmniExpress-12v1-1_B (Illumina, San Diego, CA, USA) platform, and one at deCODE (Reykjavik, Iceland), genotyped on the Human OmniExpress-12v1_H (Illumina, San Diego, CA, USA) platform. Genotypes were assigned according to the standard Illumina protocol in GenomeStudio software, version V2011.1 [13]. Quality control and imputation were performed using Ricopili [14]. The batches were merged with PLINK [15].

### Rating scales

#### Adult ADHD self-report scale

The Adult ADHD Self-Report Scale (ASRS) was developed as a screening tool to assess symptoms of ADHD in adults [16]. It includes 18 items rating symptoms of inattention, hyperactivity, and impulsivity during the past six months, based on the Diagnostic and Statistical Manual of Mental Disorders 4th edition (DSM-4) criteria for ADHD [16]. Symptom severity is reported on a 5-point Likert-type scale from 0 (never) to 4 (very often), with a range of 0–72 points.

A shorter version, the ASRS-screener, was later developed. This version has six items with a range of 0–24 points and is the official ADHD-screening tool of the World Health Organization [16]. Both the English and Norwegian translations have internal consistency and convergent validity [3, 16, 17].

In our main analyses we employed the full ASRS as a predictor, whereas in the sensitivity analyses we used the ASRS-screener as a predictor.

### The wender utah rating scale

The Wender Utah Rating Scale (WURS) retrospectively rates the frequency and severity of ADHD symptoms in childhood [18]. The WURS is based on the Utah criteria, which has a broader perspective on ADHD symptomatology than the DSM-4 criteria, including items addressing emotional regulation and conduct problems [19]. It originally included 61 items, but was condensed to the 25 items that best distinguished between cases and controls [18]. For this study we used the 25 item-version.

Participants responded to the 25 items on a Likert-type 5-point scale on a scale from 0 (not at all/very slightly) to 4 (very much), with a range of 0-100 points. Both the original English version and the Norwegian translation of the WURS have been shown to have greater diagnostic accuracy than the ASRS [3, 17].

### Calculation of polygenic scores

Polygenic scores (PGSs) were calculated using summary statistics from the 2023 ADHD Working Group of the Psychiatric Genomics Consortium GWAS meta-analysis, with the Bergen sample excluded [5]. Genotypes and GWAS summary statistics underwent quality control (QC) as outlined in Choi et al. 2022, with the additional steps described by the QC protocol for PRS-CS [6, 20]. Variants with minor allele frequency greater than 0.01, less than 5% missingness and Hardy Weinberg equilibrium of *p* > 10^− 10^ were included in the analyses. The European reference panel from the 1000 Genomes Project and default settings were used to run PRS-CS [21]. Next, ADHD PGSs were calculated with the score function in PLINK, using weights generated by PRS-CS [5, 15, 20]. The scores were residualized on the first five principal components (PCs) and batch, and standardized.

### Family history

To investigate the family history of ADHD, participants were asked whether they had first degree family members with a diagnosis of ADHD, with possible answers being “yes”, “no” and “uncertain”. Participants who responded with “uncertain” were coded as “no”, while participants with missing data were excluded from this sub-analysis.

### Statistical analyses

Eight multivariable logistic regression models were constructed with ADHD diagnosis as the outcome and different combinations of WURS-score, ASRS-score, and ADHD PGS as predictors. The base model included sex and age as predictors.

Likelihood Ratio (LR) tests were performed for the hierarchical models with and without ADHD PGS, to investigate if the ADHD PGS contributed additional information alongside the rating scales. The entire nonhierarchical set of models was also compared by their Akaike Information Criterion (AIC) and Lee R^2^ to determine whether the PGS provided the models with better model fit and phenotypic variance explained [22, 23].

AIC compares nonhierarchical models by their model fit and number of predictors to select the optimal model which best describes the data [22]. It penalizes additional predictors and prioritizes simpler models if the difference in model fit between simple and complex models is small. We used the AIC corrected for small sample sizes (AICc) [22].

The Lee R^2^ is a better coefficient of determination for genetic profile analysis with binary outcomes than pseudo-R^2^ measures based on the likelihood function, as these do not give appropriate interpretations when measuring goodness-of-fit of linear predictors of models on the scale of liability [23–25]. We elected to calculate the weighted R^2^ on the logit liability scale with a population prevalence of 2.58% [26].

As the ASRS screener is commonly used to screen for ADHD, we wanted to investigate whether the ADHD PGS contributed additional information to the ASRS-screener. We therefore repeated the analyses with the ASRS screener score as a predictor in a supplementary analysis.

In clinical interviews, patients are routinely asked for family history of ADHD, which captures some genetic liability for ADHD, and we wanted to investigate whether the ADHD PGS contributed additional information to self-reported ADHD family history. We therefore repeated the analyses with self-reported ADHD family history as a covariate as a supplementary analysis.

We also performed sensitivity analyses to investigate whether the ADHD PGS was associated with the other predictors of interest. For these analyses, we constructed three linear regression models with ASRS-score, ASRS screener-score and WURS-score as continuous outcomes with the ADHD PGS as predictor and a logistic regression model with ADHD family history as a binary outcome, with the ADHD PGS as a predictor. Sex and age were included as covariates in all models.

Analyses were conducted in GenomeStudio and R version 4.3.0 [27]. The AICcmodavg package was used to perform the AIC analysis, and the lmtest package was used to perform LR tests [28, 29]. An R Markdown document with the code used in this analysis can be found in Additional File 1.

## Results

The sample included 576 cases (51.2% female) and 530 controls (61.9% female), for a total sample size of 1106 participants (56.3% female). Demographic and clinical information for this sample is presented in Table 1. The control sample contained significantly more females than the case sample, and ADHD cases were significantly older than the controls (Table 1). Mean ASRS score, WURS score, and ADHD PGS Z-score were significantly higher in the ADHD group (Table 1).

For a more detailed table of clinical information in men and women, and in those with and without family history of ADHD, see Table S1 and Table S2 respectively in Additional File 2.

**Table 1:** Sample characteristics

	ADHD	Control	Degrees of freedom^a^	*p* value^a^
**N**	576	530		
**Sex**				
Female	295 (51.2%)	328 (61.9%)	1	0.0004
Male	281 (48.8%)	202 (38.1%)		
**Age in years, mean (SD)**	34.3 (10.2)	28.1 (6.76)	1004.1	< 0.0001
**Family history of ADHD**				
Yes	220 (38.2%)	19 (3.6%)	1	< 0.0001
Missing	7 (1.2%)	0 (0%)		
**Mean ASRS score (SD)**	44.8 (12.9)	22.7 (9.52)	1015.4	< 0.0001
**Mean WURS score (SD)**	56.6 (18.1)	16.7 (13.5)	949.5	< 0.0001
**Mean PGS (SD)**	0.111 (1.01)	-0.133 (0.97)	1102.4	< 0.0001

a. Degrees of freedom and p-values refers to the degrees of freedom and results of statistical tests comparing the groups, t-tests for continuous variables and Chi-square tests for categorical variables. ASRS: adult ADHD self-report scale, PGS: polygenic score, WURS: Wender-Utah rating scale

### Likelihood ratio test

When testing each nested pair of models with and without ADHD PGS using LR tests, we found that the PGS contributed significantly better model fit to the base model (*p* < 0.0001), the ASRS-only model (*p* = 0.0381), the WURS-only model (*p* = 0.0048), and the full model (*p* = 0.0103). The results of the LR tests for all nested pairs of models are presented in Table 2.

**Table 2:** Results of LR tests comparing models with and without PGS

	Delta chi-square [1]	*p*
PGS vs. base model^a^	15.7	< 0.001
ASRS + PGS vs. ASRS	4.3	0.0381
WURS + PGS vs. WURS	7.95	0.0048
ASRS + WURS + PGS vs. ASRS + WURS	6.58	0.0103

a. The base model includes sex and age. ASRS: adult ADHD self-report scale, PGS: polygenic score, WURS: Wender-Utah rating scale

The ADHD PGS also provided significantly better model fit to the ASRS-screener-model (*p* = 0.016), see Table S3 in Additional file 2. When self-reported ADHD family history was included in the models, we found that the PGS contributed significantly better model fit to all models, except for the ASRS only model (as well as the ASRS-screener model as assessed in the sensitivity analyses).

For the full results of likelihood ratio tests when family history of ADHD was included in the base model see Table S4 in Additional file 2.

### Akaike information criterion

When comparing the AICc of the different models, we found that the models that included the ADHD PGS had better model fit compared to the corresponding models without the ADHD PGS. For the full list of AICc, see Table 3. For a comparison of each pair of hierarchical models with and without PGS, see Tables S5, S6, S7 and S8 in the Additional File 2.

**Table 3:** Comparison of logistic regression models by AICc

	K	AICc	ΔAICc
ASRS + WURS + PGS	6	474.96	-
ASRS + WURS	5	479.52	4.56
WURS + PGS	5	523.69	48.73
WURS	4	529.62	54.66
ASRS + PGS	5	774.79	299.83
ASRS	4	777.07	302.11
PGS	4	1374.57	899.61
Base model^a^	3	1388.27	913.31

a. The base model includes sex and age. K is the number of estimated parameters in the model. ΔAICc is the difference in AICc of the best-fit model and the respective models. AICc: Akaike Information Criterion corrected, ASRS: adult ADHD self-report scale, PGS: polygenic score, WURS: Wender-Utah rating scale

The ADHD PGS also contributed with better model fit to the ASRS screener (ΔAICc = 3.78), as can be seen in Table S9 in Additional file 2. When including family history in the models, we found that inclusion of the PGS only gave a better model fit compared to an examination of family history (ΔAICc = 10.02) and the WURS-only model (ΔAICc = 2.45). For the full results of AICc analyses when family history was included in the base model, see Table S10 in Additional File 2.

### Lee R^2^

We found that the ADHD PGS increased the Lee R^2^ of all the models. The model with the highest R^2^ was the model that included the ADHD PGS and both rating scales with an R^2^ of 81.402%. The PGS increased the R^2^ of the base model by 1.518% points (pp), of the ASRS model by 0.577pp, of the WURS model by 0.610pp, and of both rating scales together by 0.565pp. See Table 4 for the full list of Lee R^2^.

**Table 4:** Lee R^2^ by different logistic regression models

	Lee *R*^2^	Incremental *R*^2^ by PGS
ASRS + WURS + PGS	81.402%	0.565pp
ASRS + WURS	80.838%	---
WURS + PGS	77.936%	0.610pp
WURS	77.326%	---
ASRS + PGS	61.686%	0.577pp
ASRS	61.109%	---
PGS	17.603%	1.518pp
Base model^a^	16.085%	---

a. The base model includes sex and age. ASRS: adult ADHD self-report scale, PGS: polygenic score, pp: percentage points, WURS: Wender-Utah rating scale

We found that the ADHD PGS increased the variance explained of the ASRS screener model by 0.662pp, as seen in Table S11 in Additional file 2. Inclusion of family history of ADHD in the models attenuated the PGS’ contribution to all the diagnostic aids, with the PGS contributing 1.395pp to the base model. For the full list of R^2^ by different models when family history was included, see Table S12 in Additional file 2.

### Sensitivity analyses

In the sensitivity analyses of the ADHD PGS’ association with the predictors of interest, we found that the ADHD PGS was significantly associated with ASRS (*p* < 0.001), ASRS-screener (*p* < 0.001), WURS (*p* < 0.001), and self-reported family history of ADHD (*p* = 0.039). For the full summary of the sensitivity analyses, see Table S13 in Additional file 2.

## Discussion

Here we aimed to investigate whether an ADHD PGS could provide additional information to commonly used diagnostic aids of ADHD in distinguishing between individuals with and without ADHD. We found that the ADHD PGS contributed with additional information to self-report rating scales and family history in a clinically representative sample of adults diagnosed with ADHD. However, the PGS’ contribution to variance explained was low, suggesting that its clinical utility in diagnostics remains limited.

We also found that the WURS performed better than the ASRS, as measured by R^2^ and AICc, as has previously been reported [3, 17]. This may be because the WURS is addressing a broader range of ADHD symptoms than ASRS.

To our knowledge, this is the first study examining the contribution of the ADHD PGS to commonly used diagnostic aids for the purpose of distinguishing between individuals with and without ADHD. Previous studies have found that ADHD PGS is associated with ADHD diagnosis in adults and children, without investigating the PGS’ potential in diagnostics alongside other diagnostic tools [30, 31]. Evidence from psychiatric genetics suggests that familial risk for psychopathology and PGS for psychiatric conditions capture slightly different genetic domains and have unique predictive powers [32]. Our findings support this, as the ADHD PGS provided supplemental information to self-reported family history of ADHD in distinguishing between cases and controls. However, our findings suggest that an interrogation of family history provides more information to identify individuals with ADHD than the ADHD PGS currently does.

While our findings suggest that the current ADHD PGS is too imprecise to have practical utility in routine diagnostics, it may be useful in predicting ADHD in combination with other screening tools. PGSs for other conditions have been used alongside other information in prediction models, such as for prediction of coronary artery disease, type 2 diabetes, and depression onset in Alzheimer’s disease [33–35]. Such models demonstrate that a PGS can provide supplementary information to other clinical instruments and biomarkers, resulting in improved prediction accuracy. In addition to being used in diagnostics, a PGS could potentially be a prognostic tool and to explore genetic liability of treatment response in psychiatric disorders [36].

Prediction models have also been developed for ADHD, employing other known risk factors such as depressive symptoms, socio-economic status, and prematurity [37, 38]. One recent study aimed to include the ADHD PGS in a prediction model of ADHD in adults and found that it increased the variance explained, without significantly improving prediction [38]. However, this PGS was based on the 2019 ADHD GWAS meta-analysis, which is less accurate than the 2023 ADHD GWAS meta-analysis used in our study [5, 38, 39]. Another recent study on the prediction of the developmental trajectory of ADHD in children found that the ADHD PGS provided information independent of other ADHD risk factors (e.g. low parental education and low birth weight) [40]. While our findings show that the ADHD PGS is limited in how much additional variance it can explain alongside commonly employed diagnostic aids, it may be more useful to investigate its utility in prediction models of ADHD alongside other risk factors, such as family environment and maternal smoking during pregnancy [7, 40–42]. Notably, all of these established risk factors have a polygenic genetic risk component that partially overlaps with ADHD genetic risk [5, 43, 44]. Future studies should investigate the ADHD PGS’ contribution to prediction models of ADHD using multiple sources of information.

Our findings must be understood in the context of certain limitations. Firstly, our sample included only Norwegians, and our results may not be generalizable to other ancestries or clinical contexts. Secondly, our participants were adults, with all cases being diagnosed with persistent, childhood onset ADHD and there were no exclusion criteria. Although children and adults with ADHD share many common genetic risk variants, the results may not be generalizable to children or adolescents, and comorbidities among the patients may have affected the association of the ADHD PGS with our ADHD diagnosis outcome [45]. However, a strength of this sample is that it consists of real-life ADHD patients, who often have diverse comorbidities [46].

Thirdly, we contrasted the utility of the PGS with the utility of the ASRS and WURS, which are useful aids in the diagnosis of ADHD, but are not necessary or sufficient to diagnose ADHD. Additionally, both the ASRS and WURS have been demonstrated to have factor structures that are more accurate in discriminating between ADHD and controls. However, in this study we decided not to use these factor structures, as the full scores are more representative of how these tools are commonly used in the clinic [16–18, 47, 48]. Finally, our study was not designed to test the utility of the ADHD PGS in a clinical diagnostic setting. However, since our results indicate only a small contribution to total variance explained, it is reasonable to believe that the ADHD PGS does not currently have clinical utility in diagnostics, despite its statistical significance.

## Conclusions

We found that the ADHD PGS contributed with additional information to commonly used self-reported clinical rating scales and self-reported family history of ADHD in distinguishing between ADHD cases and controls. However, the increase in variance explained was small and the current ADHD PGS is unlikely to have diagnostic value compared to well-established rating scales. Still, the ADHD PGS could prove useful in prediction models in combination with other types of information. Future studies should examine the utility of ADHD PGS in prediction of ADHD, and the diagnostic utility of ADHD PGS should be continually investigated as larger ADHD GWAS meta-analyses are published and new computational tools are developed.

### Electronic supplementary material

Below is the link to the electronic supplementary material.

Supplementary Material 1

Supplementary Material 2

Supplementary Material 3

## Data Availability

The datasets used and analyzed during the current study are available from the corresponding author on reasonable request.

## Abbreviations

ASRS: Adult ADHD Self-Report Scale
AIC: Akaike information criterion
AICc: Akaike information criterion corrected for small sample sizes
ADHD: Attention-deficit/hyperactivity disorder
DSM-4: Diagnostic and Statistical Manual of Mental Disorders 4th edition
GWAS: Genome-Wide Association Study
LR: Likelihood Ratio
Pp: Percentage Points
PGS: Polygenic Score
QC: Quality control
WURS: Wender Utah Rating Scale
