# Supplementary material for: Using polygenic scores in combination with symptom rating scales to identify attention-deficit/hyperactivity disorder
Source: BMC Psychiatry. 2024 Jun 27;24:471. doi: 10.1186/s12888-024-05925-7 (PMC11210094; doi:10.1186/s12888-024-05925-7)
Supplement: Supplementary file 1 — Supplementary Material 1 [file 12888_2024_5925_MOESM1_ESM.pdf]

Additional File 1

André Høberg

2024-04-09

Load packages

First we load the necessary packages.

```
library(tidyverse)
library(haven)
library(readxl)
library(dplyr)
library(data.table)
library(magrittr)
library(foreign)
library(skimr)
library(haven)
library(AICcmodavg)
library(broom)
library(fmsb)
library(flexmix)
library(lmtest)
library(forcats)
library(ggthemes)
```

Genotype quality control and polygenic score calculation

First we will quality control (QC) the base data and target data, to facilitate PGS calculation.

QC of the base data includes the following steps: -Checking effect allele -Checking file transfer

Effect allele It is important to check if the GWAS gives which allele is the effect allele.

```
base_GWAS <-fread("Data/daner_ADHD_meta_iPSYCH_deCODE_PGC_woBergen_v4-QCed_ADHD2022Demontis")
head(base_GWAS)
```

```
##    CHR        SNP        BP A1 A2 FRQ_A_38396 FRQ_U_186641 Weighted_MAF  INFO
## 1:   2 rs13424679 219184199  A  G       0.359        0.346        0.359 0.998
## 2:   2  rs4144852 215923509  G  T       0.403        0.393        0.403 1.000
## 3:   2 rs62187973 213529368  A  C       0.915        0.918        0.085 0.986
## 4:   2 rs71430232 217662995  C  T       0.942        0.943        0.058 0.993
## 5:   2  rs1831029 215725280  G  A       0.572        0.564        0.428 0.998
## 6:   2 rs73071680 211984041  A  G       0.962        0.958        0.038 0.997
##          OR     SE       P ngt    Direction HetISqt HetDf  HetPVa   Nca    Nco
## 1: 1.00844 0.0096 0.38250   0 -++---++--++    52.8    11 0.01595 38396 186641
## 2: 0.97756 0.0095 0.01672   0 +++-+-++--++    16.1    11 0.28580 38396 186641
## 3: 1.04081 0.0168 0.01747   0 ++++-+++-+--    11.3    11 0.33440 38396 186641
## 4: 1.01319 0.0195 0.50080   0 -+--++--+++-     0.0    11 0.63450 38396 186641
## 5: 1.00622 0.0095 0.51420   0 -+++--+-+-++     0.0    11 0.76660 38396 186641
```

```
## 6: 1.01959 0.0251 0.44070   0 +++-++----++     0.0    11 0.67860 38396 186641
##    Neff_half
## 1:  51327.96
## 2:  51327.96
## 3:  51327.96
## 4:  51327.96
## 5:  51327.96
## 6:  51327.96
```

Standard GWAS QC Both target and base data should go thorugh standard GWAS QC. This involves removing Single Nucleotide Polymorphisms (SNPs) with low Minor Allele Frequency (MAF) & low imputation information (INFO)

```
GWAS_base_QC_MAF_INFO <- base_GWAS[INFO > 0.9]
```

SNPs with low MAF and low INFO have already been excluded

Duplicate SNPs If there are mistakes made during the lab processes, there may be duplications of SNPs. There should only be one of every SNP in the base data.

```
ADHD_GWAS_unique_SNPs <- duplicated(GWAS_base_QC_MAF_INFO$SNP)
```

Duplicates have already been removed.

Ambiguous SNPs Ambiguous SNPs should be removed.

```
ADHD_GWAS_QCd <- subset(GWAS_base_QC_MAF_INFO,
                          !(A1 == "A" & A2 == "T"|
                              A1 == "T" & A2 == "A"|
                              A1 == "G" & A2 == "C"|
                              A1 == "C" & A2 == "G"))
```

This has also already been performed. Now we have removed ambiguous SNPs, and we have 5 749 033 SNPs left.

We want to perform QC now with the PRS-cs checklist, which includes these additional parameters: -Insert chromosome and base pair position from reference -Check allele match between sumstats and reference -Remove SNPs with reference MAF<0.01 -Remove SNPs with MAF difference between sumstats and reference >0.2 -Remove SNPs with out-of-bounds p-values (>1 o <0) -Remove SNPs with N < (90th percentile N)/2

First we load the reference dataset:

```
reference_panel <- fread("Data/ldblk_1kg_eur/snpinfo_1kg_hm3")

head(reference_panel)
```

```
##    CHR        SNP     BP A1 A2     MAF
## 1:   1  rs3131972 752721  A  G 0.16100
## 2:   1  rs3131969 754182  A  G 0.12820
## 3:   1  rs1048488 760912  C  T 0.16000
## 4:   1 rs12562034 768448  A  G 0.09245
## 5:   1  rs4040617 779322  G  A 0.11830
## 6:   1  rs4970383 838555  A  C 0.23860
```

Then we join the GWAS and reference dataset together, by their rs-number:

```
ref_gwas <- left_join(reference_panel,
                      ADHD_GWAS_QCd,
                      by = 'SNP')
head(ref_gwas)
```

```
##    CHR.x        SNP   BP.x A1.x A2.x     MAF CHR.y BP.y A1.y A2.y FRQ_A_38396
## 1:     1  rs3131972 752721    A    G 0.16100    NA   NA <NA> <NA>          NA
## 2:     1  rs3131969 754182    A    G 0.12820    NA   NA <NA> <NA>          NA
## 3:     1  rs1048488 760912    C    T 0.16000    NA   NA <NA> <NA>          NA
## 4:     1 rs12562034 768448    A    G 0.09245    NA   NA <NA> <NA>          NA
## 5:     1  rs4040617 779322    G    A 0.11830    NA   NA <NA> <NA>          NA
## 6:     1  rs4970383 838555    A    C 0.23860    NA   NA <NA> <NA>          NA
##    FRQ_U_186641 Weighted_MAF INFO OR SE  P ngt Direction HetISqt HetDf HetPVa
## 1:           NA           NA   NA NA NA NA  NA      <NA>      NA    NA     NA
## 2:           NA           NA   NA NA NA NA  NA      <NA>      NA    NA     NA
## 3:           NA           NA   NA NA NA NA  NA      <NA>      NA    NA     NA
## 4:           NA           NA   NA NA NA NA  NA      <NA>      NA    NA     NA
## 5:           NA           NA   NA NA NA NA  NA      <NA>      NA    NA     NA
## 6:           NA           NA   NA NA NA NA  NA      <NA>      NA    NA     NA
##    Nca Nco Neff_half
## 1:  NA  NA        NA
## 2:  NA  NA        NA
## 3:  NA  NA        NA
## 4:  NA  NA        NA
## 5:  NA  NA        NA
## 6:  NA  NA        NA
```

```r
sum(is.na(ref_gwas$CHR.y))
```

```
## [1] 102028
```

Now we remove the missing from ref_gwas:

```r
ref_gwas_full <- na.omit(ref_gwas)

head(ref_gwas_full)
```

```
##    CHR.x        SNP    BP.x A1.x A2.x     MAF CHR.y    BP.y A1.y A2.y
## 1:     1  rs6687776 1030565    T    C 0.16600     1 1030565    C    T
## 2:     1  rs6678318 1030633    A    G 0.16600     1 1030633    G    A
## 3:     1  rs6671356 1040026    C    T 0.13420     1 1040026    T    C
## 4:     1  rs6604968 1041700    G    A 0.13520     1 1041700    A    G
## 5:     1 rs12726255 1049950    G    A 0.13420     1 1049950    A    G
## 6:     1 rs10907175 1130727    C    A 0.08449     1 1130727    A    C
##    FRQ_A_38396 FRQ_U_186641 Weighted_MAF  INFO      OR     SE      P ngt
## 1:       0.841        0.836        0.159 0.985 0.99035 0.0134 0.4704   7
## 2:       0.841        0.837        0.159 0.985 0.99074 0.0134 0.4876   1
## 3:       0.864        0.866        0.136 0.998 0.98452 0.0138 0.2576   5
## 4:       0.864        0.865        0.136 0.995 0.98403 0.0137 0.2401   1
## 5:       0.863        0.865        0.137 0.985 0.98403 0.0140 0.2508   4
## 6:       0.909        0.913        0.091 0.995 0.99700 0.0159 0.8525   6
##       Direction HetISqt HetDf HetPVa   Nca    Nco Neff_half
## 1: +-+++?+??-+-       0     8 0.7665 36963 184077  49598.31
## 2: +-+++?+??-+-       0     8 0.7892 36963 184077  49598.31
## 3: -+----+-?---       0    10 0.9978 37772 184886  50407.31
## 4: -+----+-?---       0    10 0.9965 37772 184886  50407.31
## 5: -+----+-?---       0    10 0.9946 37772 184886  50407.31
## 6: +-----+?++-+       0    10 0.4437 37696 185941  50627.96
```

We now have 1 070 726 SNPs.

Checking allele match.

```r
ref_gwas_allele_m <- ref_gwas_full[(A1.x == A1.y & A2.x == A2.y) |
                                     (A1.x == A2.y & A2.x == A1.y)]

head(ref_gwas_allele_m)
```

```
##    CHR.x        SNP    BP.x A1.x A2.x     MAF CHR.y    BP.y A1.y A2.y
## 1:     1  rs6687776 1030565    T    C 0.16600     1 1030565    C    T
## 2:     1  rs6678318 1030633    A    G 0.16600     1 1030633    G    A
## 3:     1  rs6671356 1040026    C    T 0.13420     1 1040026    T    C
## 4:     1  rs6604968 1041700    G    A 0.13520     1 1041700    A    G
## 5:     1 rs12726255 1049950    G    A 0.13420     1 1049950    A    G
## 6:     1 rs10907175 1130727    C    A 0.08449     1 1130727    A    C
##    FRQ_A_38396 FRQ_U_186641 Weighted_MAF  INFO      OR     SE      P ngt
## 1:       0.841        0.836        0.159 0.985 0.99035 0.0134 0.4704   7
## 2:       0.841        0.837        0.159 0.985 0.99074 0.0134 0.4876   1
## 3:       0.864        0.866        0.136 0.998 0.98452 0.0138 0.2576   5
## 4:       0.864        0.865        0.136 0.995 0.98403 0.0137 0.2401   1
## 5:       0.863        0.865        0.137 0.985 0.98403 0.0140 0.2508   4
## 6:       0.909        0.913        0.091 0.995 0.99700 0.0159 0.8525   6
##       Direction HetISqt HetDf HetPVa   Nca    Nco Neff_half
## 1: +-+++?+??-+-       0     8 0.7665 36963 184077  49598.31
## 2: +-+++?+??-+-       0     8 0.7892 36963 184077  49598.31
## 3: -+----+-?---       0    10 0.9978 37772 184886  50407.31
## 4: -+----+-?---       0    10 0.9965 37772 184886  50407.31
## 5: -+----+-?---       0    10 0.9946 37772 184886  50407.31
## 6: +-----+?++-+       0    10 0.4437 37696 185941  50627.96
```

All the alleles matched.

Now excluding SNPs with reference MAF < 0.01 and a difference in MAF between reference panel and sumstats >0.2:

```r
ref_gwas_MAF <- ref_gwas_allele_m[!MAF < 0.01 & !MAF - FRQ_A_38396 > 0.2]
```

0 SNPs were excluded.

Removing SNPs with invalid p-values:

```r
ref_gwas_p <- ref_gwas_MAF[P<1 & P>0]
```

46 variants had invalid p-values.

Now excluding SNPs with N less than the 90th percentile N divided by 2:

0 SNPs were excluded.

Now we want to only include the relevant variables, and change their names back to norm:

```r
ADHD_GWAS_final_QC <- subset(ADHD_GWAS_QC,
                             select = c("CHR.x", "SNP", "BP.x", "A1.x", "A2.x",
                                        "MAF", "FRQ_A_38396", "FRQ_U_186641",
                                        "INFO", "OR", "SE", "P", "ngt",
                                        "Direction", "HetISqt", "HetDf", "HetPVa",
                                        "Nca", "Nco", "Neff_half")
                             ) %>%
  rename('CHR' = 'CHR.x',
         'BP' = 'BP.x',
         'A1' = 'A1.x',
```

```
         'A2' = 'A2.x')

summary(ADHD_GWAS_final_QC)
```

```
##       CHR              SNP                  BP                  A1
##  Min.   : 1.000   Length:1018622     Min.   :    18674   Length:1018622
##  1st Qu.: 4.000   Class :character   1st Qu.: 32902672   Class :character
##  Median : 8.000   Mode  :character   Median : 70199370   Mode  :character
##  Mean   : 8.604                      Mean   : 79363971
##  3rd Qu.:13.000                      3rd Qu.:115207019
##  Max.   :22.000                      Max.   :249191732
##       A2                 MAF           FRQ_A_38396      FRQ_U_186641
##  Length:1018622     Min.   :0.01093   Min.   :0.0101   Min.   :0.0109
##  Class :character   1st Qu.:0.12430   1st Qu.:0.4380   1st Qu.:0.4380
##  Mode  :character   Median :0.23760   Median :0.6830   Median :0.6830
##                     Mean   :0.24611   Mean   :0.6291   Mean   :0.6289
##                     3rd Qu.:0.36480   3rd Qu.:0.8520   3rd Qu.:0.8520
##                     Max.   :0.50000   Max.   :0.9900   Max.   :0.9890
##       INFO              OR               SE                  P
##  Min.   :0.9160   Min.   :0.8624   Min.   :0.00810   Min.   :0.0000
##  1st Qu.:0.9920   1st Qu.:0.9902   1st Qu.:0.00980   1st Qu.:0.1756
##  Median :0.9970   Median :0.9999   Median :0.01110   Median :0.4311
##  Mean   :0.9939   Mean   :0.9999   Mean   :0.01321   Mean   :0.4501
##  3rd Qu.:0.9990   3rd Qu.:1.0094   3rd Qu.:0.01440   3rd Qu.:0.7122
##  Max.   :1.0000   Max.   :1.1704   Max.   :0.04970   Max.   :0.9999
##       ngt           Direction            HetISqt          HetDf
##  Min.   : 0.000   Length:1018622     Min.   : 0.00   Min.   : 2.00
##  1st Qu.: 2.000   Class :character   1st Qu.: 0.00   1st Qu.:11.00
##  Median : 4.000   Mode  :character   Median : 0.00   Median :11.00
##  Mean   : 3.856                      Mean   :11.21   Mean   :10.58
##  3rd Qu.: 6.000                      3rd Qu.:21.00   3rd Qu.:11.00
##  Max.   :11.000                      Max.   :86.70   Max.   :11.00
##      HetPVa              Nca              Nco            Neff_half
##  Min.   :0.0000007   Min.   :34176   Min.   :175141   Min.   :45996
##  1st Qu.:0.2396000   1st Qu.:38396   1st Qu.:186641   1st Qu.:51328
##  Median :0.4869000   Median :38396   Median :186641   Median :51328
##  Mean   :0.4906999   Mean   :38175   Mean   :186077   Mean   :51031
##  3rd Qu.:0.7392000   3rd Qu.:38396   3rd Qu.:186641   3rd Qu.:51328
##  Max.   :1.0000000   Max.   :38396   Max.   :186641   Max.   :51328
```

```
fwrite(ADHD_GWAS_final_QC,
       "Data/2023_ADHD_GWAS_QCd_wo_deCODE.qc",
       sep="\t")
```

Now we have produced a file which is the QCd GWAS sum stats.

Now we will QC the target dataset.

First we will merge the two target datasets, named Broad and deCODE, in order to create a PGS for both groups. We know that the Broad sample has many invalid rsIDs and duplicates, so we will remove these first, then we will begin merging the datasets.

```
bergen_broad_bim <- fread("Data/genotype/BROAD/bergen-broad.bim") %>%
  setnames(., colnames(.), c("CHR", "SNP", "CM", "BP", "B.A1", "B.A2")) %>%
  .[,c("B.A1", "B.A2"):=list(toupper(B.A1), toupper(B.A2))] %>%
  mutate(SNP = ifelse(grepl(pattern = ":",
```

```
                      x = SNP,
                      fixed = TRUE)
                == TRUE,
                substr(SNP,
                       1,
                       17),
                SNP),
  SNP = ifelse(grepl(pattern = ":",
                      x = SNP,
                      fixed = TRUE)
                == TRUE,
                substr(SNP,
                       1,
                       16),
                SNP),
  SNP = ifelse(grepl(pattern = ":",
                     x = SNP,
                     fixed = TRUE)
                == TRUE,
                substr(SNP,
                       1,
                       15),
                SNP),
  SNP = ifelse(grepl(pattern = ":",
                      x = SNP,
                      fixed = TRUE)
                == TRUE,
                substr(SNP,
                       1,
                       14),
                SNP),
  SNP = ifelse(grepl(pattern = ":",
                      x = SNP,
                      fixed = TRUE)
                == TRUE,
                substr(SNP,
                       1,
                       13),
                SNP),
  SNP = ifelse(grepl(pattern = ":",
                      x = SNP,
                      fixed = TRUE)
                == TRUE,
                substr(SNP,
                       1,
                       12),
                SNP),
  SNP = ifelse(grepl(pattern = ":",
                      x = SNP,
                      fixed = TRUE)
                == TRUE,
                substr(SNP,
                       1,
```

```
                    11),
                SNP),
    SNP = ifelse(grepl(pattern = ":",
                       x = SNP,
                       fixed = TRUE)
                == TRUE,
                substr(SNP,
                    1,
                    10),
                SNP),
    SNP = ifelse(grepl(pattern = ":",
                       x = SNP,
                       fixed = TRUE)
                == TRUE,
                substr(SNP,
                    1,
                    9),
                SNP),
    SNP = ifelse(grepl(pattern = ":",
                       x = SNP,
                       fixed = TRUE)
                == TRUE,
                substr(SNP,
                    1,
                    8),
                SNP),
    SNP = ifelse(grepl(pattern = ":",
                      x = SNP,
                      fixed = TRUE)
                == TRUE,
                substr(SNP,
                    1,
                    7),
                SNP),
    SNP = ifelse(grepl(pattern = ":",
                       x = SNP,
                       fixed = TRUE)
                == TRUE,
                substr(SNP,
                    1,
                    6),
                SNP),
    SNP = ifelse(grepl(pattern = ":",
                       x = SNP,
                       fixed = TRUE)
                == TRUE,
                substr(SNP,
                    1,
                    5),
                SNP),
    SNP = ifelse(grepl(pattern = ":",
                       x = SNP,
                       fixed = TRUE)
```

```
                      == TRUE,
                      substr(SNP,
                             1,
                             4),
                      SNP),
         SNP = ifelse(grepl(pattern = ":",
                            x = SNP,
                            fixed = TRUE)
                      == TRUE,
                      substr(SNP,
                             1,
                             3),
                      SNP),
         SNP = ifelse(grepl(pattern = ":",
                            x = SNP,
                            fixed = TRUE)
                      == TRUE,
                      substr(SNP,
                             1,
                             2),
                      SNP),
         SNP = ifelse(grepl(pattern = ":",
                            x = SNP,
                            fixed = TRUE)
                      == TRUE,
                      substr(SNP,
                             1,
                             1),
                      SNP))


fwrite(bergen_broad_bim,
       "Data/bergen-broad_09.bim",
       col.names = F,
       sep = "\t")
```

Then we run the following code with Plink2 to remove duplicates: ./plink2 –bfile Data/bergen-broad_09 –rm-dup exclude all –make-bed –out Data/bergen-broad_09_no_dup_SNPs

Now we will create a SNP-list with the common SNPs between deCODE and Broad:

```
bergen_broad_bim <- fread("Data/bergen-broad_09_no_dup_SNPs.bim") %>%
  setnames(., colnames(.), c("CHR", "SNP", "CM", "BP", "B.A1", "B.A2")) %>%
  subset(select = c("SNP", "CHR", "BP"))

summary(bergen_broad_bim)
```

```
##      SNP                 CHR               BP
##  Length:4989817     Min.   : 1.000   Min.   :     2564
##  Class :character   1st Qu.: 4.000   1st Qu.: 36351030
##  Mode  :character   Median : 8.000   Median : 72816066
##                     Mean   : 8.484   Mean   : 81712331
##                     3rd Qu.:12.000   3rd Qu.:117237584
##                     Max.   :22.000   Max.   :249211619
```

```r
head(bergen_broad_bim)
```

```
##            SNP CHR     BP
## 1: exm2216283   1 564766
## 2:  rs3094315   1 752566
## 3:  rs3131972   1 752721
## 4:  rs3131971   1 752894
## 5:  rs2073814   1 753474
## 6:  rs3115859   1 754503
```

```r
#The Broad dataset includes 4 989 817 SNPs

#Now we read in sumstats data
deCODE_bim <- fread("Data/genotype/DECODE/best-guess-maf.bim") %>%
  setnames(., colnames(.), c("CHR", "SNP", "CM", "BP", "A1", "A2")) %>%
  subset(select = c("SNP", "CHR", "BP"))

summary(deCODE_bim)
```

```
##      SNP                 CHR              BP
##  Length:7542776     Min.   : 1.000   Min.   :      828
##  Class :character   1st Qu.: 4.000   1st Qu.: 33361082
##  Mode  :character   Median : 7.000   Median : 69961072
##                     Mean   : 8.517   Mean   : 79232259
##                     3rd Qu.:12.000   3rd Qu.:114379442
##                     Max.   :22.000   Max.   :249232965
```

```r
head(deCODE_bim)
```

```
##            SNP CHR     BP
## 1: chr1:60249   1  60249
## 2: chr1:73490   1  73490
## 3:  rs3094315   1 752566
## 4:  rs3131972   1 752721
## 5:  rs3131971   1 752894
## 6:  rs2073814   1 753474
```

```r
#The deCODE dataset includes 7 542 776 SNPs

#Now we merge the .bim files and write out their common SNPs
merged_info <- inner_join(bergen_broad_bim,
                          deCODE_bim,
                          by = c("SNP", "CHR","BP"))

fwrite(merged_info[, c("SNP")],
       "Data/2023_11_07_bergen-broad_deCODE_overlap.SNPlist",
       col.names = F,
       sep = "\t")
```

We found that they shared 4 781 290 SNPs

We use the following code to merge the .bed datasets: ./plink2 –bfile Data/bergen-broad_09_no_dup_SNPs –extract Data/2023_11_07_bergen-broad_deCODE_overlap.SNPlist –make-bed –out Data/2023_11_07_bergen-broad_deCODE_overlap

./plink2 –bfile Data/genotype/DECODE/best-guess-maf –extract Data/2023_11_07_bergen-broad_deCODE_overlap.SNPlist –make-bed –out Data/2023_11_07_deCODE_bergen-broad_overlap

./plink –bfile Data/2023_11_07_deCODE_bergen-broad_overlap –bmerge Data/2023_11_07_bergen-broad_deCODE_overlap –out Data/2023_10_31_BBD_merged_rem

Looking at the misssnp variants, we found that ~10 000 variants had invalid alleles (More than 1 allele, and alleles with invalid nucleotides). We will exclude these from the analysis.

```
missnp <- fread("Data/2023_10_31_BBD_merged_rem.missnp",
             col.names = c("SNP")
             )

head(missnp)
```

```
##           SNP
## 1: rs10002114
## 2: rs10011527
## 3: rs10039975
## 4:    rs10045
## 5: rs10048775
## 6: rs10064136
```

```
missnp_broad <- inner_join(missnp,
                           bergen_broad_bim,
                           by = "SNP")

head(missnp_broad)
```

```
##           SNP CHR        BP
## 1: rs10002114   4  66339432
## 2: rs10011527   4 184507881
## 3: rs10039975   5  29687969
## 4:    rs10045   3 122144952
## 5: rs10048775   2 153479712
## 6: rs10064136   5   7891101
```

./plink2 –bfile Data/bergen-broad_09_no_dup_SNPs –extract Data/2023_11_07_bergen-broad_deCODE_overlap.SNPlist –exclude Data/2023_10_31_BBD_merged_rem.missnp –make-bed –out Data/2023_11_07_bergen-broad_deCODE_overlap_rem

./plink2 –bfile Data/genotype/DECODE/best-guess-maf –extract Data/2023_11_07_bergen-broad_deCODE_overlap.SNPlist –exclude Data/2023_10_31_BBD_merged_rem.missnp –make-bed –out Data/2023_11_07_deCODE_bergen-broad_overlap_rem

Now we try merging again: ./plink –bfile Data/2023_11_07_deCODE_bergen-broad_overlap_rem –bmerge Data/2023_11_07_bergen-broad_deCODE_overlap_rem –out Data/2023_11_07_BBD_merged

Now we can perform QC of the merged dataset. We received 132 same-positionw arnings, but with 4 769 047 SNPs, this should not be too much of a problem. The merged file includes 1 245 individuals.

Sample size and duplicates It is recommended that PGS analyses are conducted on sample sizes of >100.

```
fam <- fread("Data/2023_11_07_BBD_merged.fam")

nrow(fam)
```

```
## [1] 1245
```

The sample contains 1245 individuals.

Duplicate SNPs First we want to remove duplicates: ./plink2 –bfile Data/2023_11_07_BBD_merged –rm-dup exclude-all –make-bed –out Data/2023_11_07_BBD_merged_no_dup_SNPs There were no duplicate

SNPs.

Standard GWAS QC We will put the data through standard GWAS QC, which involves: -Removing SNPs with low MAF -Removing SNPs with low imputation information -Removing individuals with low genotyping rate -Removing SNPs out of Hardy-Weinberg equilibrium We do this by writing the following code in the terminal: ./plink –bfile Data/2023_11_07_BBD_merged –maf 0.01 –hwe 1e-10 –geno 0.05 –mind 0.05 –write-snplist –make-just-fam –out Data/2023_11_07_BBD_merged_QC –must-have-sex –noweb

4 769 047 variants and 1245 individuals passed filters and QC, among them 676 cases and 553 controls.

Now we want to prune away SNPs which are highly correlated, to avoid over-fitting We do this with the following code in the terminal: ./plink –bfile Data/2023_11_07_BBD_merged –keep Data/2023_11_07_BBD_merged_QC.fam –extract Data/2023_11_07_BBD_merged_QC.snplist –indep-pairwise 200 50 0.25 –out Data/2023_11_07_BBD_merged_QC

4 568 675 variants were pruned out, leaving 200 372

This produces two files: broad_QC.prune.in and broad_QC.prune.out broad.prune.in contains the SNPs that are less than 0.25 correlated Now we will use this file to get a measure of heterozygozity in our sample, using plink: ./plink –bfile Data/2023_11_07_BBD_merged –extract Data/2023_11_07_BBD_merged_QC.prune.in –keep Data/2023_11_07_BBD_merged_QC.fam –het –out Data/2023_11_07_BBD_merged_QC

This produces 2023_11_07_BBD_merged.het, which holds each participant's F-coefficient, a measure of heterozygosity. We will remove the individuals with F-coefficients greater than 3 SDs from the mean:

```
QC_het <- fread("Data/2023_11_07_BBD_merged_QC.het")
QC_het_valid <- QC_het[F<=mean(F) + 3*sd(F) &
                         F>=mean(F) - 3*sd(F)]
```

4 individuals were removed.

Now we write out the IDs of the valid observations:

```
fwrite(QC_het_valid[,c("FID", "IID")],
       "Data/2023_11_07_BBD_merged_QC.valid.sample",
       sep="\t")
```

Mismatched SNPs SNPs that do not match between base data and target data may be resolved by flipping the SNPs to their complementary SNPs. First we load the .bim file, the GWAS summary statistics and QC SNP list into R.

```
bim <- fread("Data/2023_11_07_BBD_merged.bim") %>%
  setnames(colnames(.),
           c("CHR",
             "SNP",
             "CM",
             "BP",
             "B.A1",
             "B.A2")) %>%
  .[,c("B.A1", "B.A2"):=list(toupper(B.A1), toupper(B.A2))]

head(bergen_broad_bim)
```

```
##           SNP CHR     BP
## 1: exm2216283   1 564766
## 2:  rs3094315   1 752566
## 3:  rs3131972   1 752721
## 4:  rs3131971   1 752894
## 5:  rs2073814   1 753474
```

```
## 6:  rs3115859   1 754503
```

Now we read in sumstats data.

```
sumstats <- fread("Data/2023_ADHD_GWAS_QCd_wo_deCODE.qc") %>%
  .[, c("A1", "A2") := list(toupper(A1), toupper(A2)
                            )
    ] %>%
  mutate(CHR = as.numeric(as.character(CHR)
                            )
         )
```

Now we read in QCed SNPs.

```
SNP_QC <- fread("Data/2023_11_07_BBD_merged_QC.snplist",
                header = F)
```

Now we identify the SNPs that require strand flipping. First we merge sumstats with the target sample and filter out QC'd SNPs:

```
merged_info <- merge(bim,
                     sumstats,
                     by = c("SNP",
                            "CHR",
                            "BP")
                     ) %>%
  .[SNP %in% SNP_QC[,V1]]
```

Now we create a function to calculate the complementary allele

```
complement <- function(x){
  switch(x,
         "A" = "T",
         "T" = "A",
         "C" = "G",
         "G" = "C",
         return(NA)
  )
}
```

Now we find the SNPs that have the same alleles across base and target samples:

```
matched_merged_info <- merged_info[A1 == B.A1 & A2 == B.A2, SNP]
```

615 204 SNPs have the same alleles across base and target samples.

Now we identify the SNPs that have corresponding alleles between base and target:

```
corresponding_SNPs <- merged_info[sapply(B.A1, complement) == A1 &
                                    sapply(B.A2, complement) == A2,
                                  SNP]
```

No SNPs were complementary between base and target

Now we will identify the SNPs that require recoding in the target:

```
recode_SNPs <- merged_info[B.A1==A2 & B.A2==A1, SNP]
```

14 422 SNPs require recoding.

Now we update the .bim files:

```
bim[SNP %in% recode_SNPs,
    c("B.A1", "B.A2") := list(B.A2, B.A1)
    ]
```

Identify the SNPs that need recoding & complement:

```
com_recode <- merged_info[sapply(B.A1, complement) == A2 &
                            sapply(B.A2, complement) == A1,
                          SNP
                          ]
```

No SNPs required recoding and complement.

Now we write the updated .bim file

```
fwrite(bim[, c("SNP", "B.A1")],
       "Data/2023_11_07_BBD_merged_QC.a1",
       col.names = F,
       sep = "\t")
```

Now we identify SNPs that have different alleles in target and base datasets.

```
mismatch <- bim[!(SNP %in% matched_merged_info|
                    SNP %in% corresponding_SNPs|
                    SNP %in% com_recode |
                    SNP %in% recode_SNPs),
                SNP]
```

4 139 421 SNPs were mismatched. This is because the European 1KG reference panel includes few SNPs.

```
fwrite(as.list(mismatch),
       "Data/2023_11_07_BBD_merged_QC.mismatch",
       col.names = F,
       sep = "\t")
```

We can then use bergen_broad.a1 to update the A1 alleles

Relatedness Any closely related individuals have to be removed in order for the results to be generalizable We do this in plink with the following code: ./plink –bfile Data/2023_11_07_BBD_merged –extract Data/2023_11_07_BBD_merged_QC.prune.in –keep Data/2023_11_07_BBD_merged_QC.het.valid.sample –rel-cutoff 0.125 –out Data/2023_11_07_BBD_merged_QC

77 individuals were excluded by a relatedness cutoff of 0.125

Making the final QC'd file Now we make final QC'd target file, using plink ./plink –bfile Data/2023_11_07_BBD_merged –make-bed –keep Data/2023_11_07_BBD_merged_QC.fam –out Data/2023_11_07_BBD_merged_QC –extract Data/2023_11_07_BBD_merged_QC.snplist –exclude Data/2023_11_07_BBD_merged_QC.mismatch –a1-allele Data/2023_11_07_BBD_merged_QC.a1

With this done we have made a QC'd set of files and gone through the list of steps to make the target data eligible for PGS calculation.

Calculating the first 5 Principal Components (PCs) for the BROAD-data, to be used as covariates in analyses. ./plink –bfile Data/2023_11_07_BBD_merged_QC –extract Data/2023_11_07_BBD_merged_QC.prune.in –pca 5 –out Data/2023_11_07_BBD_merged_QC

Now the genetic data is ready to be used in PGS calculation. To calculate the PGS with PRS-CS, we have to change the format of the sumimary statistics. It needs to have the format: rs-id, A1, A2, OR, P

```
sumstats_PRS_cs <- subset(sumstats,
                          select= c("SNP",
```

```
                                    "A1",
                                    "A2",
                                    "OR",
                                    "P")
                          )
fwrite(sumstats_PRS_cs,
       "Data/genotype/sumstats.txt",
       sep = "\t")
```

We run PRS-cs with standard parameters: python PRScs-master/PRScs.py –ref_dir=Data/ldblk_1kg_eur –bim_prefix=Data/2023_11_07_BBD_merged_QC –sst_file=Data/genotype/sumstats.txt –n_gwas=225036 –out_dir=Data/2023_11_07_BBD_merged_QC

This gives us chromosome, rs ID, base position, A1, A2 and posterior effect size estimate for each SNP

Now we have to merge the individual chromosome data:

```
chr1 <- fread("Data/2023_11_07_BBD_merged_QC_pst_eff_a1_b0.5_phiauto_chr1.txt")
chr2 <- fread("Data/2023_11_07_BBD_merged_QC_pst_eff_a1_b0.5_phiauto_chr2.txt")
chr3 <- fread("Data/2023_11_07_BBD_merged_QC_pst_eff_a1_b0.5_phiauto_chr3.txt")
chr4 <- fread("Data/2023_11_07_BBD_merged_QC_pst_eff_a1_b0.5_phiauto_chr4.txt")
chr5 <- fread("Data/2023_11_07_BBD_merged_QC_pst_eff_a1_b0.5_phiauto_chr5.txt")
chr6 <- fread("Data/2023_11_07_BBD_merged_QC_pst_eff_a1_b0.5_phiauto_chr6.txt")
chr7 <- fread("Data/2023_11_07_BBD_merged_QC_pst_eff_a1_b0.5_phiauto_chr7.txt")
chr8 <- fread("Data/2023_11_07_BBD_merged_QC_pst_eff_a1_b0.5_phiauto_chr8.txt")
chr9 <- fread("Data/2023_11_07_BBD_merged_QC_pst_eff_a1_b0.5_phiauto_chr9.txt")
chr10 <- fread("Data/2023_11_07_BBD_merged_QC_pst_eff_a1_b0.5_phiauto_chr10.txt")
chr11 <- fread("Data/2023_11_07_BBD_merged_QC_pst_eff_a1_b0.5_phiauto_chr11.txt")
chr12 <- fread("Data/2023_11_07_BBD_merged_QC_pst_eff_a1_b0.5_phiauto_chr12.txt")
chr13 <- fread("Data/2023_11_07_BBD_merged_QC_pst_eff_a1_b0.5_phiauto_chr13.txt")
chr14 <- fread("Data/2023_11_07_BBD_merged_QC_pst_eff_a1_b0.5_phiauto_chr14.txt")
chr15 <- fread("Data/2023_11_07_BBD_merged_QC_pst_eff_a1_b0.5_phiauto_chr15.txt")
chr16 <- fread("Data/2023_11_07_BBD_merged_QC_pst_eff_a1_b0.5_phiauto_chr16.txt")
chr17 <- fread("Data/2023_11_07_BBD_merged_QC_pst_eff_a1_b0.5_phiauto_chr17.txt")
chr18 <- fread("Data/2023_11_07_BBD_merged_QC_pst_eff_a1_b0.5_phiauto_chr18.txt")
chr19 <- fread("Data/2023_11_07_BBD_merged_QC_pst_eff_a1_b0.5_phiauto_chr19.txt")
chr20 <- fread("Data/2023_11_07_BBD_merged_QC_pst_eff_a1_b0.5_phiauto_chr20.txt")
chr21 <- fread("Data/2023_11_07_BBD_merged_QC_pst_eff_a1_b0.5_phiauto_chr21.txt")
chr22 <- fread("Data/2023_11_07_BBD_merged_QC_pst_eff_a1_b0.5_phiauto_chr22.txt")

chr_list <- list(chr1, chr2, chr3, chr4, chr5, chr6, chr7, chr8, chr9, chr10,
                 chr11, chr12, chr13, chr14, chr15, chr16, chr17, chr18, chr19,
                 chr20, chr21, chr22)

Chr_full <- chr_list %>%
  reduce(full_join)
```

```
## Joining with `by = join_by(V1, V2, V3, V4, V5, V6)`
## Joining with `by = join_by(V1, V2, V3, V4, V5, V6)`
## Joining with `by = join_by(V1, V2, V3, V4, V5, V6)`
## Joining with `by = join_by(V1, V2, V3, V4, V5, V6)`
## Joining with `by = join_by(V1, V2, V3, V4, V5, V6)`
## Joining with `by = join_by(V1, V2, V3, V4, V5, V6)`
## Joining with `by = join_by(V1, V2, V3, V4, V5, V6)`
## Joining with `by = join_by(V1, V2, V3, V4, V5, V6)`
```

```
## Joining with `by = join_by(V1, V2, V3, V4, V5, V6)`
## Joining with `by = join_by(V1, V2, V3, V4, V5, V6)`
## Joining with `by = join_by(V1, V2, V3, V4, V5, V6)`
## Joining with `by = join_by(V1, V2, V3, V4, V5, V6)`
## Joining with `by = join_by(V1, V2, V3, V4, V5, V6)`
## Joining with `by = join_by(V1, V2, V3, V4, V5, V6)`
## Joining with `by = join_by(V1, V2, V3, V4, V5, V6)`
## Joining with `by = join_by(V1, V2, V3, V4, V5, V6)`
## Joining with `by = join_by(V1, V2, V3, V4, V5, V6)`
## Joining with `by = join_by(V1, V2, V3, V4, V5, V6)`
## Joining with `by = join_by(V1, V2, V3, V4, V5, V6)`
## Joining with `by = join_by(V1, V2, V3, V4, V5, V6)`
## Joining with `by = join_by(V1, V2, V3, V4, V5, V6)`
```

```
fwrite(Chr_full,
       "Results/2023_11_07_BBD_merged_QC_SNP",
       sep = "\t")
```

Then we use the –score flag in plink to calculate PGS with the posterior effect sizes. ./plink –bfile Data/2023_11_07_BBD_merged_QC –score Results/2023_11_07_BBD_merged_QC_SNP 2 5 6 sum –out Results/2023_11_07_BBD_merged_QC

Readying data

With the PGS calculated, we will ready the data we will use for analysis. We will start by merging the necessary phenotype files, then the PGS file, and finally we will merge these to create a dataset for analysis.

First for the phenotype. The 2005 dataset is split in two different datasets with slightly different variables. We subset the necessary variables from each dataset, and then merge them:

```
ADHD_data_del1 <- read_sav("Data/phenotype/ADHD_data_del1_korr.gender_2019.sav")

ADHD_data_del2 <- read_sav("Data/phenotype/ADHDdata_part2.sav") %>%
  mutate(ADHD_id = Genetisk_nr)

head(ADHD_data_del2)
```

```
## # A tibble: 6 x 223
##   Nummer Genetisk_nr Date  Grtilh    Grtilh2   Kjonn   Fodselsaar Alder Tvilling
##    <dbl> <chr>       <chr> <dbl+lbl> <dbl+lbl> <dbl+l>      <dbl> <dbl> <dbl+lb>
## 1    649 A-649       ""    1 [pas]   1 [pas]   2 [man~         NA    NA NA
## 2    654 A-654       ""    1 [pas]   1 [pas]   1 [kvi~       1963    45  2 [Nei]
## 3    656 A-656       ""    1 [pas]   1 [pas]   1 [kvi~       1962    47  2 [Nei]
## 4    657 A-657       ""    1 [pas]   1 [pas]   2 [man~       1963    46  2 [Nei]
## 5    658 A-658       ""    1 [pas]   1 [pas]   2 [man~       1968    42  2 [Nei]
## 6    662 A-662       ""    1 [pas]   1 [pas]   1 [kvi~       1987    23  2 [Nei]
## # i 214 more variables: EtniskBakgrunn <dbl+lbl>, utdanning <dbl+lbl>,
## #   arbeid <dbl+lbl>, ADHDbarn1 <dbl+lbl>, ADHDbarn2 <dbl+lbl>,
## #   Behbarn1 <dbl+lbl>, Behbarn2 <dbl+lbl>, dysleksi <dbl+lbl>,
## #   epilepsi <dbl+lbl>, migrene <dbl+lbl>, astma <dbl+lbl>,
## #   angstdepr <dbl+lbl>, autTicTourAspberg <dbl+lbl>, PU <dbl+lbl>,
## #   bipolar <dbl+lbl>, alkohol <dbl+lbl>, rus <dbl+lbl>, rusprobl <dbl+lbl>,
## #   annenpsyk <dbl+lbl>, psykbeh <dbl+lbl>, spiseforstyrr <dbl+lbl>, ...
```

```
ADHD_pheno_merged <- full_join(ADHD_data_del1,
                               ADHD_data_del2) %>%
```

```r
  mutate(ASRSscreen = ASRS1 + ASRS2 + ASRS3 + ASRS4 + ASRS5 + ASRS6,
         ASRS_tot = ASRSscreen + ASRS7 + ASRS8 + ASRS9 + ASRS10 + ASRS11 +
           ASRS12 + ASRS13 + ASRS14 + ASRS15 + ASRS16 + ASRS17 + ASRS18,
         WURS_tot = WURS1 + WURS2 + WURS3 + WURS4 + WURS5 + WURS6 + WURS7 +
           WURS8 + WURS9 + WURS10 + WURS11 + WURS12 + WURS13 + WURS14 + WURS15 +
           WURS16 + WURS17 + WURS18 + WURS19 + WURS20 + WURS21 + WURS22 +
           WURS23 + WURS24 + WURS25,
         FamADHD = ifelse(FamADHD == 2,
                          3,
                          FamADHD),
         FamADHD = ifelse(FamADHD == 1,
                          2,
                          FamADHD),
         FamADHD = ifelse(FamADHD == 3,
                          1,
                          FamADHD),
         FamADHD = as.ordered(FamADHD),
         Kjonn = as.factor(Kjonn),
         Grtilh = as.factor(Grtilh)
         ) %>%
  subset(select = c("ADHD_id", "ASRSscreen", "ASRS_tot", "WURS_tot",
                    "Kjonn", "Alder", "Grtilh", "FamADHD")
         )
```

```
## Joining with `by = join_by(Nummer, ADHD_id, Grtilh, Grtilh2, Kjonn, Fodselsaar,
## Alder, utdanning, arbeid, ADHDbarn1, ADHDbarn2, Behbarn1, Behbarn2, dysleksi,
## epilepsi, migrene, astma, angstdepr, autTicTourAspberg, PU, bipolar, alkohol,
## rus, rusprobl, annenpsyk, psykbeh, FamADHD, FamDysleksi, FamEpilepsi,
## FamMigrene, FamAstma, FamAngstDepr, FamAutTicTou, FamPU, FamBipolar,
## FamAlkohol, FamRus, FamPsykAnnen, ASRS1, ASRS2, ASRS3, ASRS4, ASRS5, ASRS6,
## ASRS7, ASRS8, ASRS9, ASRS10, ASRS11, ASRS12, ASRS13, ASRS14, ASRS15, ASRS16,
## ASRS17, ASRS18, WURS1, WURS2, WURS3, WURS4, WURS5, WURS6, WURS7, WURS8, WURS9,
## WURS10, WURS11, WURS12, WURS13, WURS14, WURS15, WURS16, WURS17, WURS18, WURS19,
## WURS20, WURS21, WURS22, WURS23, WURS24, WURS25, `filter_$`, Alder_2022)`
```

Then we load the PGS file by merging together the PGS file, the ID files and the PCs. We also residualize the PGS on the first 5 PCs and normalize it, for use in T-tests.

```r
PGS <- read.table("Results/2023_11_03_BBD.profile",
                  header = TRUE) %>%
  rename(SCORE = "SCORESUM")

BROAD_IDs <- read_excel("Data/genotype/BROAD/broad-qc-id.xlsx") %>%
  rename(IID = "Collaborator Participant ID",
         ADHD_id = "Bergen ID")%>%
  mutate(batch = 0)

deCODE_IDs <- fread("Data/genotype/DECODE/gwas-gender-age-id") %>%
  rename(IID = "V5",
         ADHD_id = "V7") %>%
  subset(select = c("IID",
                    "ADHD_id")
         ) %>%
  mutate(batch = 1)
```

```r
IDs <- full_join(x = BROAD_IDs,
                 y = deCODE_IDs)
```

```
## Joining with `by = join_by(IID, ADHD_id, batch)`
```

```r
covar <- read.table("Data/2023_11_07_BBD_merged_QC.eigenvec") %>%
  rename(IID = "V2")

merg_PGS <- full_join(x = IDs,
                      y = covar,
                      by = "IID") %>%
  inner_join(x = .,
             y = PGS,
             by = "IID") %>%
  subset(is.na(V3) == FALSE &
           is.na(SCORE) == FALSE &
           is.na(V4) == FALSE &
           is.na(V5) == FALSE &
           is.na(V6) == FALSE &
           is.na(V7) == FALSE &
           is.na(batch) == FALSE,
         select = c("ADHD_id", "SCORE", "IID", "V3", "V4", "V5", "V6", "V7",
                    "batch"),
         drop = TRUE ) %>%
  mutate(batch = as.factor(batch)
         )

PGS_residuals <- residuals(lm(formula = SCORE ~ V3 + V4 + V5 + V6 + V7 + batch,
                              data = merg_PGS)
                           )

merg_PGS <- mutate(merg_PGS,
                   res_SCORE = PGS_residuals,
                   res_SCORE = as.numeric(res_SCORE)
                   )

res_SD <- sd(merg_PGS$res_SCORE)
mean_SD <- mean(merg_PGS$res_SCORE,
                na.rm = TRUE)

merg_PGS <- merg_PGS %>%
  mutate(Z_res_SCORE = (res_SCORE - mean_SD) / res_SD
         )
```

Then we merge the PGS file and the phenotypic file:

```r
ADHD_PGS <- left_join(ADHD_pheno_merged,
                      merg_PGS,
                      by = "ADHD_id")%>%
  subset(!is.na(Z_res_SCORE) &
           !is.na(Alder) &
           !is.na(Kjonn)
         )

summary(ADHD_PGS)
```

```
##    ADHD_id           ASRSscreen      ASRS_tot       WURS_tot      Kjonn
##  Length:1106        Min.   : 0.00   Min.   : 0.00   Min.   :  0.00   1:623
##  Class :character   1st Qu.: 7.00   1st Qu.:21.00   1st Qu.: 13.00   2:483
##  Mode  :character   Median :11.00   Median :32.00   Median : 35.00
##                     Mean   :11.95   Mean   :34.14   Mean   : 36.82
##                     3rd Qu.:17.00   3rd Qu.:48.00   3rd Qu.: 57.00
##                     Max.   :55.00   Max.   :77.00   Max.   :108.00
##                     NA's   :22      NA's   :37      NA's   :85
##      Alder       Grtilh  FamADHD       SCORE          IID
##  Min.   :17.00   1:576   1   :860   Min.   :-3.566   Length:1106
##  1st Qu.:24.00   2:530   2   :239   1st Qu.:-2.930   Class :character
##  Median :30.00           NA's:  7   Median :-2.795   Mode  :character
##  Mean   :31.33                      Mean   :-2.796
##  3rd Qu.:37.00                      3rd Qu.:-2.660
##  Max.   :67.00                      Max.   :-2.150
##
##        V3                   V4                   V5
##  Min.   :-0.1939120   Min.   :-0.2007690   Min.   :-0.2290790
##  1st Qu.:-0.0056514   1st Qu.:-0.0088020   1st Qu.:-0.0068040
##  Median : 0.0005322   Median :-0.0015723   Median :-0.0007020
##  Mean   :-0.0001304   Mean   :-0.0005114   Mean   :-0.0002378
##  3rd Qu.: 0.0064410   3rd Qu.: 0.0056357   3rd Qu.: 0.0058684
##  Max.   : 0.2090290   Max.   : 0.1790180   Max.   : 0.2308210
##
##        V6                   V7              batch     res_SCORE
##  Min.   :-0.2562350   Min.   :-0.2167610   0:729   Min.   :-0.7791975
##  1st Qu.:-0.0068486   1st Qu.:-0.0071208   1:377   1st Qu.:-0.1382993
##  Median :-0.0008872   Median :-0.0009574           Median :-0.0004578
##  Mean   :-0.0004848   Mean   :-0.0003117           Mean   :-0.0012123
##  3rd Qu.: 0.0050654   3rd Qu.: 0.0055563           3rd Qu.: 0.1343403
##  Max.   : 0.1908560   Max.   : 0.1838440           Max.   : 0.6313683
##
##   Z_res_SCORE
##  Min.   :-3.836627
##  1st Qu.:-0.680961
##  Median :-0.002254
##  Mean   :-0.005969
##  3rd Qu.: 0.661467
##  Max.   : 3.108743
##
```

Checking demographic information

Looking at a general summary of the whole dataset. The following code is the source for Table 1.

```
summary(ADHD_PGS$Grtilh)
```

```
##   1   2
## 576 530
```

Now creating two different datasets for Cases and Controls

```
ADHD_Control <- subset(ADHD_PGS,
                       Grtilh == 2)

ADHD_Case <- subset(ADHD_PGS,
```

```r
                    Grtilh == 1)
```

Now looking at the age variable:

```r
mean(ADHD_Control$Alder)
```

```
## [1] 28.05094
```

```r
sd(ADHD_Control$Alder)
```

```
## [1] 6.756587
```

```r
summary(ADHD_Control$Alder)
```

```
##    Min. 1st Qu.  Median    Mean 3rd Qu.    Max.
##   18.00   22.00   27.00   28.05   34.00   59.00
```

```r
mean(ADHD_Case$Alder)
```

```
## [1] 34.34028
```

```r
sd(ADHD_Case$Alder)
```

```
## [1] 10.22948
```

```r
summary(ADHD_Case$Alder)
```

```
##    Min. 1st Qu.  Median    Mean 3rd Qu.    Max.
##   17.00   25.00   34.00   34.34   41.00   67.00
```

```r
t.test(Alder ~ Grtilh,
       data = ADHD_PGS)
```

```
##
##  Welch Two Sample t-test
##
## data:  Alder by Grtilh
## t = 12.153, df = 1004.1, p-value < 2.2e-16
## alternative hypothesis: true difference in means between group 1 and group 2 is not equal to 0
## 95 percent confidence interval:
##  5.273831 7.304838
## sample estimates:
## mean in group 1 mean in group 2
##        34.34028        28.05094
```

Now looking at the sex:

```r
summary(ADHD_Control$Kjonn)
```

```
##   1   2
## 328 202
```

```r
prop.table(table(ADHD_Control$Kjonn)
           )
```

```
##
##         1         2
## 0.6188679 0.3811321
```

```r
summary(ADHD_Case$Kjonn)
```

```
##   1   2
```

```
## 295 281
```

```r
prop.table(table(ADHD_Case$Kjonn)
           )
```

```
##
##         1         2
## 0.5121528 0.4878472
```

```r
Grtilh_Gender <- table(ADHD_PGS$Grtilh, ADHD_PGS$Kjonn)
chisq.test(Grtilh_Gender)
```

```
##
##  Pearson's Chi-squared test with Yates' continuity correction
##
## data:  Grtilh_Gender
## X-squared = 12.348, df = 1, p-value = 0.0004414
```

Now we look at WURS-scores:

```r
mean(ADHD_Control$WURS_tot,
     na.rm = TRUE)
```

```
## [1] 16.72978
```

```r
sd(ADHD_Control$WURS_tot,
   na.rm = TRUE)
```

```
## [1] 13.54177
```

```r
summary(ADHD_Control$WURS_tot)
```

```
##    Min. 1st Qu.  Median    Mean 3rd Qu.    Max.    NA's
##    0.00    7.00   13.00   16.73   21.00   91.00      23
```

```r
mean(ADHD_Case$WURS_tot,
     na.rm = TRUE)
```

```
## [1] 56.64202
```

```r
sd(ADHD_Case$WURS_tot,
   na.rm = TRUE)
```

```
## [1] 18.12917
```

```r
summary(ADHD_Case$WURS_tot)
```

```
##    Min. 1st Qu.  Median    Mean 3rd Qu.    Max.    NA's
##    7.00   43.00   56.00   56.64   70.00  108.00      62
```

```r
t.test(WURS_tot ~ Grtilh,
       data = ADHD_PGS)
```

```
##
##  Welch Two Sample t-test
##
## data:  WURS_tot by Grtilh
## t = 39.89, df = 949.49, p-value < 2.2e-16
## alternative hypothesis: true difference in means between group 1 and group 2 is not equal to 0
## 95 percent confidence interval:
##  37.94867 41.87581
## sample estimates:
```

```
## mean in group 1 mean in group 2
##        56.64202        16.72978
```

Now we look at ASRS-score:

```r
mean(ADHD_Control$ASRS_tot,
     na.rm = TRUE)
```

```
## [1] 22.67573
```

```r
sd(ADHD_Control$ASRS_tot,
   na.rm = TRUE)
```

```
## [1] 9.517836
```

```r
summary(ADHD_Control$ASRS_tot)
```

```
##    Min. 1st Qu.  Median    Mean 3rd Qu.    Max.    NA's
##    0.00   16.00   21.00   22.68   27.50   62.00      15
```

```r
mean(ADHD_Case$ASRS_tot,
     na.rm = TRUE)
```

```
## [1] 44.79422
```

```r
sd(ADHD_Case$ASRS_tot,
   na.rm = TRUE)
```

```
## [1] 12.90699
```

```r
summary(ADHD_Case$ASRS_tot)
```

```
##    Min. 1st Qu.  Median    Mean 3rd Qu.    Max.    NA's
##    5.00   37.00   47.00   44.79   54.00   77.00      22
```

```r
t.test(ASRS_tot ~ Grtilh,
       data = ADHD_PGS)
```

```
##
##  Welch Two Sample t-test
##
## data:  ASRS_tot by Grtilh
## t = 32.039, df = 1015.4, p-value < 2.2e-16
## alternative hypothesis: true difference in means between group 1 and group 2 is not equal to 0
## 95 percent confidence interval:
##  20.76379 23.47320
## sample estimates:
## mean in group 1 mean in group 2
##        44.79422        22.67573
```

Now looking at PGS:

```r
mean(ADHD_Control$Z_res_SCORE,
     na.rm = TRUE)
```

```
## [1] -0.1334426
```

```r
sd(ADHD_Control$Z_res_SCORE,
   na.rm = TRUE)
```

```
## [1] 0.9650466
```

```r
summary(ADHD_Control$Z_res_SCORE)
```

```
##    Min. 1st Qu.  Median    Mean 3rd Qu.    Max. 
## -3.5365 -0.8105 -0.1264 -0.1334  0.4783  3.1087
```

```r
mean(ADHD_Case$Z_res_SCORE,
     na.rm = TRUE)
```

```
## [1] 0.1113242
```

```r
sd(ADHD_Case$Z_res_SCORE,
   na.rm = TRUE)
```

```
## [1] 1.009126
```

```r
summary(ADHD_Case$Z_res_SCORE)
```

```
##    Min. 1st Qu.  Median    Mean 3rd Qu.    Max. 
## -3.8366 -0.5479  0.1288  0.1113  0.7837  3.0523
```

```r
t.test(Z_res_SCORE ~ Grtilh,
       data = ADHD_PGS)
```

```
## 
##  Welch Two Sample t-test
## 
## data:  Z_res_SCORE by Grtilh
## t = 4.1225, df = 1102.4, p-value = 4.028e-05
## alternative hypothesis: true difference in means between group 1 and group 2 is not equal to 0
## 95 percent confidence interval:
##  0.1282702 0.3612635
## sample estimates:
## mean in group 1 mean in group 2 
##       0.1113242      -0.1334426
```

Looking at familial history of ADHD:

```r
summary(ADHD_Case$FamADHD)
```

```
##    1    2 NA's 
##  349  220    7
```

```r
prop.table(table(ADHD_Case$FamADHD)
           )
```

```
## 
##         1         2 
## 0.6133568 0.3866432
```

```r
summary(ADHD_Control$FamADHD)
```

```
##   1   2 
## 511  19
```

```r
prop.table(table(ADHD_Control$FamADHD)
           )
```

```
## 
##          1          2 
## 0.96415094 0.03584906
```

```r
Grtilh_FamADHD <- table(ADHD_PGS$Grtilh,
                        ADHD_PGS$FamADHD)
chisq.test(Grtilh_FamADHD)
```

```
##
##  Pearson's Chi-squared test with Yates' continuity correction
##
## data:  Grtilh_FamADHD
## X-squared = 196.37, df = 1, p-value < 2.2e-16
```

Now looking at clinical information and comparing them between sexes. The following code is the source for Table S1:

```r
Male <- subset(ADHD_PGS,
                       Kjonn == 2)

Female <- subset(ADHD_PGS,
                    Kjonn == 1)

#Now we look at WURS-score
mean(Male$WURS_tot,
     na.rm = TRUE)
```

```
## [1] 39.49336
```

```r
sd(Male$WURS_tot,
   na.rm = TRUE)
```

```
## [1] 25.58179
```

```r
summary(Male$WURS_tot)
```

```
##    Min. 1st Qu.  Median    Mean 3rd Qu.    Max.    NA's
##    0.00   16.00   38.50   39.49   60.00  100.00      31
```

```r
mean(Female$WURS_tot,
     na.rm = TRUE)
```

```
## [1] 34.70123
```

```r
sd(Female$WURS_tot,
   na.rm = TRUE)
```

```
## [1] 25.42094
```

```r
summary(Female$WURS_tot)
```

```
##    Min. 1st Qu.  Median    Mean 3rd Qu.    Max.    NA's
##     0.0    12.0    30.0    34.7    55.0   108.0      54
```

```r
t.test(WURS_tot ~ Kjonn,
       data = ADHD_PGS)
```

```
##
##  Welch Two Sample t-test
##
## data:  WURS_tot by Kjonn
## t = -2.9814, df = 964.72, p-value = 0.002941
## alternative hypothesis: true difference in means between group 1 and group 2 is not equal to 0
## 95 percent confidence interval:
```

```
##  -7.946434 -1.637831
## sample estimates:
## mean in group 1 mean in group 2
##        34.70123        39.49336
```

```r
#Now we look at ASRS-score
mean(Male$ASRS_tot,
     na.rm = TRUE)
```

```
## [1] 34.1383
```

```r
sd(Male$ASRS_tot,
   na.rm = TRUE)
```

```
## [1] 15.66426
```

```r
summary(Male$ASRS_tot)
```

```
##    Min. 1st Qu.  Median    Mean 3rd Qu.    Max.    NA's
##    0.00   21.00   33.00   34.14   48.00   71.00      13
```

```r
mean(Female$ASRS_tot,
     na.rm = TRUE)
```

```
## [1] 34.13856
```

```r
sd(Female$ASRS_tot,
   na.rm = TRUE)
```

```
## [1] 16.05704
```

```r
summary(Female$ASRS_tot)
```

```
##    Min. 1st Qu.  Median    Mean 3rd Qu.    Max.    NA's
##    1.00   21.00   31.00   34.14   49.00   77.00      24
```

```r
t.test(ASRS_tot ~ Kjonn,
       data = ADHD_PGS)
```

```
##
##  Welch Two Sample t-test
##
## data:  ASRS_tot by Kjonn
## t = 0.00027296, df = 1018.3, p-value = 0.9998
## alternative hypothesis: true difference in means between group 1 and group 2 is not equal to 0
## 95 percent confidence interval:
##  -1.914852  1.915384
## sample estimates:
## mean in group 1 mean in group 2
##        34.13856        34.13830
```

```r
#Now we look at residualized PGS
mean(Male$Z_res_SCORE,
     na.rm = TRUE)
```

```
## [1] -0.009295794
```

```r
sd(Male$Z_res_SCORE,
   na.rm = TRUE)
```

```
## [1] 1.027951
```

```r
summary(Male$Z_res_SCORE)
```

```
##      Min.   1st Qu.    Median      Mean   3rd Qu.      Max. 
## -3.836627 -0.783173  0.006206 -0.009296  0.701237  2.800241
```

```r
mean(Female$Z_res_SCORE,
     na.rm = TRUE)
```

```
## [1] -0.003390026
```

```r
sd(Female$Z_res_SCORE,
   na.rm = TRUE)
```

```
## [1] 0.9701446
```

```r
summary(Female$Z_res_SCORE)
```

```
##     Min.  1st Qu.   Median     Mean  3rd Qu.     Max. 
## -3.53654 -0.63889 -0.01416 -0.00339  0.62932  3.10874
```

```r
t.test(Z_res_SCORE ~ Kjonn,
       data = ADHD_PGS)
```

```
## 
##  Welch Two Sample t-test
## 
## data:  Z_res_SCORE by Kjonn
## t = 0.09711, df = 1005.8, p-value = 0.9227
## alternative hypothesis: true difference in means between group 1 and group 2 is not equal to 0
## 95 percent confidence interval:
##  -0.1134332  0.1252447
## sample estimates:
## mean in group 1 mean in group 2 
##    -0.003390026    -0.009295794
```

Now looking at clinical information and comparing them between those with and without a family history of ADHD. The following code is the source for Table S2:

```r
yes_FH <- subset(ADHD_PGS,
                 FamADHD == 2)

no_FH <- subset(ADHD_PGS,
                FamADHD == 1)

#Now we look at WURS-score
mean(yes_FH$WURS_tot,
     na.rm = TRUE)
```

```
## [1] 53.20465
```

```r
sd(yes_FH$WURS_tot,
   na.rm = TRUE)
```

```
## [1] 20.58139
```

```r
summary(yes_FH$WURS_tot)
```

```
##    Min. 1st Qu.  Median    Mean 3rd Qu.    Max.    NA's 
##     1.0    39.0    54.0    53.2    68.5   108.0      24
```

```r
mean(no_FH$WURS_tot,
     na.rm = TRUE)
```

```
## [1] 32.37267
```

```r
sd(no_FH$WURS_tot,
   na.rm = TRUE)
```

```
## [1] 24.93963
```

```r
summary(no_FH$WURS_tot)
```

```
##    Min. 1st Qu.  Median    Mean 3rd Qu.    Max.    NA's
##    0.00   11.00   25.00   32.37   52.00  100.00      55
```

```r
t.test(WURS_tot ~ FamADHD,
       data = ADHD_PGS)
```

```
##
##  Welch Two Sample t-test
##
## data:  WURS_tot by FamADHD
## t = -12.579, df = 398.45, p-value < 2.2e-16
## alternative hypothesis: true difference in means between group 1 and group 2 is not equal to 0
## 95 percent confidence interval:
##  -24.08788 -17.57608
## sample estimates:
## mean in group 1 mean in group 2
##        32.37267        53.20465
```

```r
#Now we look at ASRS-score
mean(yes_FH$ASRS_tot,
     na.rm = TRUE)
```

```
## [1] 43.6824
```

```r
sd(yes_FH$ASRS_tot,
   na.rm = TRUE)
```

```
## [1] 13.3695
```

```r
summary(yes_FH$ASRS_tot)
```

```
##    Min. 1st Qu.  Median    Mean 3rd Qu.    Max.    NA's
##    5.00   35.00   46.00   43.68   54.00   77.00       6
```

```r
mean(no_FH$ASRS_tot,
     na.rm = TRUE)
```

```
## [1] 31.44671
```

```r
sd(no_FH$ASRS_tot,
   na.rm = TRUE)
```

```
## [1] 15.48374
```

```r
summary(no_FH$ASRS_tot)
```

```
##    Min. 1st Qu.  Median    Mean 3rd Qu.    Max.    NA's
##    0.00   19.50   28.00   31.45   44.00   77.00      25
```

```r
t.test(ASRS_tot ~ FamADHD,
       data = ADHD_PGS)
```

```
##
##  Welch Two Sample t-test
##
## data:  ASRS_tot by FamADHD
## t = -11.917, df = 421.73, p-value < 2.2e-16
## alternative hypothesis: true difference in means between group 1 and group 2 is not equal to 0
## 95 percent confidence interval:
##  -14.25393 -10.21747
## sample estimates:
## mean in group 1 mean in group 2
##        31.44671        43.68240
```

```r
#Now we look at residualized PGS
mean(yes_FH$Z_res_SCORE,
     na.rm = TRUE)
```

```
## [1] 0.1359346
```

```r
sd(yes_FH$Z_res_SCORE,
   na.rm = TRUE)
```

```
## [1] 1.00851
```

```r
summary(yes_FH$Z_res_SCORE)
```

```
##    Min. 1st Qu.  Median    Mean 3rd Qu.    Max.
## -2.3336 -0.5623  0.1549  0.1359  0.7475  3.0523
```

```r
mean(no_FH$Z_res_SCORE,
     na.rm = TRUE)
```

```
## [1] -0.04221409
```

```r
sd(no_FH$Z_res_SCORE,
   na.rm = TRUE)
```

```
## [1] 0.9896106
```

```r
summary(no_FH$Z_res_SCORE)
```

```
##     Min.  1st Qu.   Median     Mean  3rd Qu.     Max.
## -3.83663 -0.70292 -0.04410 -0.04221  0.62336  3.10874
```

```r
t.test(Z_res_SCORE ~ FamADHD,
       data = ADHD_PGS)
```

```
##
##  Welch Two Sample t-test
##
## data:  Z_res_SCORE by FamADHD
## t = -2.4256, df = 374.97, p-value = 0.01576
## alternative hypothesis: true difference in means between group 1 and group 2 is not equal to 0
## 95 percent confidence interval:
##  -0.32256708 -0.03373032
## sample estimates:
## mean in group 1 mean in group 2
```

```
##     -0.04221409      0.13593462
```

Logistic regression models

Now we can run the logistic regression models and compare them by LR tests, AICc and Lee R^2. First we create all the models.

First WURS model:

```
Model_WURS <- glm(Grtilh ~ WURS_tot + Kjonn + Alder,
                  family = binomial(logit),
                  data = ADHD_PGS)

tidy(Model_WURS)
```

```
## # A tibble: 4 x 5
##   term        estimate std.error statistic  p.value
##   <chr>          <dbl>     <dbl>     <dbl>    <dbl>
## 1 (Intercept)   7.21     0.565       12.8  2.94e-37
## 2 WURS_tot     -0.129    0.00793    -16.3  5.99e-60
## 3 Kjonn2       -0.583    0.229       -2.54 1.10e- 2
## 4 Alder        -0.0809   0.0134      -6.03 1.65e- 9
```

Then the WURS and PGS model:

```
Model_WURS_PGS <- glm(Grtilh ~ WURS_tot + Z_res_SCORE + Kjonn + Alder,
                      family = binomial(logit),
                      data = ADHD_PGS)

tidy(Model_WURS_PGS)
```

```
## # A tibble: 5 x 5
##   term        estimate std.error statistic  p.value
##   <chr>          <dbl>     <dbl>     <dbl>    <dbl>
## 1 (Intercept)   7.30     0.575       12.7  6.96e-37
## 2 WURS_tot     -0.130    0.00805    -16.2  7.03e-59
## 3 Z_res_SCORE  -0.323    0.115       -2.81 5.02e- 3
## 4 Kjonn2       -0.599    0.231       -2.59 9.52e- 3
## 5 Alder        -0.0826   0.0137      -6.04 1.50e- 9
```

The ASRS model:

```
Model_ASRS <- glm(Grtilh ~ ASRS_tot + Kjonn + Alder,
                  family = binomial(logit),
                  data = ADHD_PGS)

tidy(Model_ASRS)
```

```
## # A tibble: 4 x 5
##   term        estimate std.error statistic  p.value
##   <chr>          <dbl>     <dbl>     <dbl>    <dbl>
## 1 (Intercept)   7.22     0.484       14.9  1.94e-50
## 2 ASRS_tot     -0.144    0.00853    -16.9  8.22e-64
## 3 Kjonn2       -0.794    0.187       -4.25 2.10e- 5
## 4 Alder        -0.0727   0.0112      -6.48 9.11e-11
```

The ASRS and PGS model:

```r
Model_ASRS_PGS <- glm(Grtilh ~ ASRS_tot + Z_res_SCORE + Kjonn + Alder,
                      family = binomial(logit),
                      data = ADHD_PGS)

tidy(Model_ASRS_PGS)
```

```
## # A tibble: 5 x 5
##   term        estimate std.error statistic  p.value
##   <chr>          <dbl>     <dbl>     <dbl>    <dbl>
## 1 (Intercept)   7.24     0.487       14.9  5.56e-50
## 2 ASRS_tot     -0.144    0.00857    -16.8  4.94e-63
## 3 Z_res_SCORE  -0.193    0.0933      -2.07 3.87e- 2
## 4 Kjonn2       -0.813    0.188       -4.34 1.44e- 5
## 5 Alder        -0.0734   0.0113      -6.49 8.55e-11
```

The ASRS-screener model:

```r
Model_ASRS_screen <- glm(Grtilh ~ ASRSscreen + Kjonn + Alder,
                         family = binomial(logit),
                         data = ADHD_PGS)

tidy(Model_ASRS_screen)
```

```
## # A tibble: 4 x 5
##   term        estimate std.error statistic  p.value
##   <chr>          <dbl>     <dbl>     <dbl>    <dbl>
## 1 (Intercept)   6.99      0.467      15.0  1.22e-50
## 2 ASRSscreen   -0.371     0.0222    -16.7  1.73e-62
## 3 Kjonn2       -0.729     0.179      -4.06 4.88e- 5
## 4 Alder        -0.0802    0.0109     -7.39 1.51e-13
```

The ASRS-screener and PGS model:

```r
Model_ASRS_screen_PGS <- glm(Grtilh ~ ASRSscreen + Z_res_SCORE + Kjonn + Alder,
                             family = binomial(logit),
                             data = ADHD_PGS)

tidy(Model_ASRS_screen_PGS)
```

```
## # A tibble: 5 x 5
##   term        estimate std.error statistic  p.value
##   <chr>          <dbl>     <dbl>     <dbl>    <dbl>
## 1 (Intercept)   7.01      0.471      14.9  4.23e-50
## 2 ASRSscreen   -0.370     0.0223    -16.6  1.10e-61
## 3 Z_res_SCORE  -0.217     0.0902     -2.40 1.64e- 2
## 4 Kjonn2       -0.751     0.181      -4.16 3.20e- 5
## 5 Alder        -0.0810    0.0110     -7.39 1.50e-13
```

The WURS and ASRS model:

```r
Model_WURS_ASRS <- glm(Grtilh ~ WURS_tot + ASRS_tot + Kjonn + Alder,
                       family = binomial(logit),
                       data = ADHD_PGS)
tidy(Model_WURS_ASRS)
```

```
## # A tibble: 5 x 5
##   term        estimate std.error statistic  p.value
```

```
##   <chr>           <dbl>     <dbl>     <dbl>    <dbl>
## 1 (Intercept)   8.47      0.657       12.9   5.06e-38
## 2 WURS_tot     -0.103     0.00844    -12.1   6.62e-34
## 3 ASRS_tot     -0.0716    0.0109      -6.60 4.24e-11
## 4 Kjonn2       -0.788     0.247       -3.19 1.42e- 3
## 5 Alder        -0.0709    0.0141      -5.03 4.84e- 7
```

The full model:

```
Model_WURS_ASRS_PGS <- glm(Grtilh ~ WURS_tot + ASRS_tot + Z_res_SCORE + Kjonn +
                             Alder,
                           family = binomial(logit),
                           data = ADHD_PGS)

tidy(Model_WURS_ASRS_PGS)
```

```
## # A tibble: 6 x 5
##   term        estimate std.error statistic  p.value
##   <chr>          <dbl>     <dbl>     <dbl>    <dbl>
## 1 (Intercept)   8.57      0.671       12.8   2.45e-37
## 2 WURS_tot     -0.104     0.00857    -12.2   5.31e-34
## 3 ASRS_tot     -0.0709    0.0109      -6.52 6.95e-11
## 4 Z_res_SCORE  -0.313     0.123       -2.55 1.07e- 2
## 5 Kjonn2       -0.800     0.248       -3.22 1.27e- 3
## 6 Alder        -0.0729    0.0144      -5.07 4.02e- 7
```

The PGS model:

```
Model_PGS <- glm(Grtilh ~ Z_res_SCORE + Kjonn + Alder,
                 family = binomial(logit),
                 data = ADHD_PGS)

tidy(Model_PGS)
```

```
## # A tibble: 4 x 5
##   term        estimate std.error statistic  p.value
##   <chr>          <dbl>     <dbl>     <dbl>    <dbl>
## 1 (Intercept)   2.74      0.260       10.5   7.20e-26
## 2 Z_res_SCORE  -0.261     0.0667      -3.92 8.81e- 5
## 3 Kjonn2       -0.478     0.131       -3.65 2.64e- 4
## 4 Alder        -0.0846    0.00796    -10.6   2.01e-26
```

The base model:

```
Model_Base <- glm(Grtilh ~ Kjonn + Alder,
                  family = binomial(logit),
                  data = ADHD_PGS)

tidy(Model_Base)
```

```
## # A tibble: 3 x 5
##   term        estimate std.error statistic  p.value
##   <chr>          <dbl>     <dbl>     <dbl>    <dbl>
## 1 (Intercept)   2.73      0.258       10.6   4.03e-26
## 2 Kjonn2       -0.473     0.130       -3.64 2.78e- 4
## 3 Alder        -0.0842    0.00787    -10.7   1.02e-26
```

The PGS only model:

```r
Model_PGS_only <- glm(Grtilh ~ Z_res_SCORE,
                      family = binomial(logit),
                      data = ADHD_PGS)

tidy(Model_PGS_only)
```

```
## # A tibble: 2 x 5
##   term        estimate std.error statistic   p.value
##   <chr>          <dbl>     <dbl>     <dbl>     <dbl>
## 1 (Intercept)  -0.0859    0.0607     -1.42 0.157
## 2 Z_res_SCORE  -0.251     0.0620     -4.05 0.0000503
```

Performing LR tests comparing each pair of rating scale data and PGS and rating scale. The following code is the source for Table 2.

```r
lrtest(Model_Base,
       Model_PGS)
```

```
## Likelihood ratio test
##
## Model 1: Grtilh ~ Kjonn + Alder
## Model 2: Grtilh ~ Z_res_SCORE + Kjonn + Alder
##   #Df  LogLik Df  Chisq Pr(>Chisq)
## 1   3 -691.12
## 2   4 -683.27  1 15.717  7.354e-05 ***
## ---
## Signif. codes:  0 '***' 0.001 '**' 0.01 '*' 0.05 '.' 0.1 ' ' 1
```

```r
lrtest(Model_WURS_ASRS,
       Model_WURS_ASRS_PGS)
```

```
## Likelihood ratio test
##
## Model 1: Grtilh ~ WURS_tot + ASRS_tot + Kjonn + Alder
## Model 2: Grtilh ~ WURS_tot + ASRS_tot + Z_res_SCORE + Kjonn + Alder
##   #Df  LogLik Df  Chisq Pr(>Chisq)
## 1   5 -234.73
## 2   6 -231.44  1 6.5775    0.01033 *
## ---
## Signif. codes:  0 '***' 0.001 '**' 0.01 '*' 0.05 '.' 0.1 ' ' 1
```

```r
lrtest(Model_WURS,
       Model_WURS_PGS)
```

```
## Likelihood ratio test
##
## Model 1: Grtilh ~ WURS_tot + Kjonn + Alder
## Model 2: Grtilh ~ WURS_tot + Z_res_SCORE + Kjonn + Alder
##   #Df  LogLik Df Chisq Pr(>Chisq)
## 1   4 -260.79
## 2   5 -256.81  1  7.95   0.004809 **
## ---
## Signif. codes:  0 '***' 0.001 '**' 0.01 '*' 0.05 '.' 0.1 ' ' 1
```

```r
lrtest(Model_ASRS,
       Model_ASRS_PGS)
```

```
## Likelihood ratio test
##
## Model 1: Grtilh ~ ASRS_tot + Kjonn + Alder
## Model 2: Grtilh ~ ASRS_tot + Z_res_SCORE + Kjonn + Alder
##   #Df  LogLik Df  Chisq Pr(>Chisq)
## 1   4 -384.52
## 2   5 -382.37  1 4.2986    0.03814 *
## ---
## Signif. codes:  0 '***' 0.001 '**' 0.01 '*' 0.05 '.' 0.1 ' ' 1
```

The following code is the source for Table S3:

```
lrtest(Model_ASRS_screen,
       Model_ASRS_screen_PGS)
```

```
## Likelihood ratio test
##
## Model 1: Grtilh ~ ASRSscreen + Kjonn + Alder
## Model 2: Grtilh ~ ASRSscreen + Z_res_SCORE + Kjonn + Alder
##   #Df  LogLik Df  Chisq Pr(>Chisq)
## 1   4 -407.32
## 2   5 -404.42  1 5.7984    0.01604 *
## ---
## Signif. codes:  0 '***' 0.001 '**' 0.01 '*' 0.05 '.' 0.1 ' ' 1
```

Using AIC to investigate which models fits the data best. First for the main models we are interested in. This is the source for Table 3.

```
models <- list(Model_WURS, Model_WURS_PGS, Model_ASRS, Model_ASRS_PGS,
               Model_WURS_ASRS, Model_WURS_ASRS_PGS, Model_PGS, Model_Base)

model.names <- c('WURS', 'WURS+PGS', 'ASRS', 'ASRS+PGS', 'ASRS+WURS',
                 'ASRS+WURS+PGS', 'PGS', "Base")

model_aic <- aictab(cand.set = models, modnames = model.names)

model_aic
```

```
##
## Model selection based on AICc:
##
##               K    AICc Delta_AICc AICcWt Cum.Wt      LL
## ASRS+WURS+PGS 6  474.96       0.00   0.91   0.91 -231.44
## ASRS+WURS     5  479.52       4.55   0.09   1.00 -234.73
## WURS+PGS      5  523.69      48.72   0.00   1.00 -256.81
## WURS          4  529.62      54.65   0.00   1.00 -260.79
## ASRS+PGS      5  774.79     299.83   0.00   1.00 -382.37
## ASRS          4  777.07     302.11   0.00   1.00 -384.52
## PGS           4 1374.57     899.60   0.00   1.00 -683.27
## Base          3 1388.27     913.31   0.00   1.00 -691.12
```

Then we do this for each pair of hierarchical models with and without PGS. ASRS-screener. This is the source for Tables S5, S6, S7, S8 and S9.

```
models <- list(Model_WURS_ASRS, Model_WURS_ASRS_PGS)

model.names <- c('ASRS+WURS', 'ASRS+WURS+PGS')
```

```r
model_aic <- aictab(cand.set = models, modnames = model.names)

model_aic
```

```
##
## Model selection based on AICc:
##
##                K   AICc Delta_AICc AICcWt Cum.Wt      LL
## ASRS+WURS+PGS 6 474.96       0.00   0.91   0.91 -231.44
## ASRS+WURS     5 479.52       4.55   0.09   1.00 -234.73
```

```r
models <- list(Model_WURS, Model_WURS_PGS)

model.names <- c('WURS', 'WURS+PGS')

model_aic <- aictab(cand.set = models, modnames = model.names)

model_aic
```

```
##
## Model selection based on AICc:
##
##           K   AICc Delta_AICc AICcWt Cum.Wt      LL
## WURS+PGS 5 523.69       0.00   0.95   0.95 -256.81
## WURS     4 529.62       5.93   0.05   1.00 -260.79
```

```r
models <- list(Model_ASRS, Model_ASRS_PGS)

model.names <- c('ASRS', 'ASRS+PGS')

model_aic <- aictab(cand.set = models, modnames = model.names)

model_aic
```

```
##
## Model selection based on AICc:
##
##           K   AICc Delta_AICc AICcWt Cum.Wt      LL
## ASRS+PGS 5 774.79       0.00   0.76   0.76 -382.37
## ASRS     4 777.07       2.28   0.24   1.00 -384.52
```

```r
models <- list(Model_Base, Model_PGS)

model.names <- c('Base', 'PGS')

model_aic <- aictab(cand.set = models, modnames = model.names)

model_aic
```

```
##
## Model selection based on AICc:
##
##      K    AICc Delta_AICc AICcWt Cum.Wt      LL
## PGS  4 1374.57        0.0      1      1 -683.27
## Base 3 1388.27       13.7      0      1 -691.12
```

```r
models <- list(Model_ASRS_screen, Model_ASRS_screen_PGS)

model.names <- c('ASRS screen', 'ASRS screen + PGS')

model_aic <- aictab(cand.set = models, modnames = model.names)

model_aic
```

```
##
## Model selection based on AICc:
##
##                   K   AICc Delta_AICc AICcWt Cum.Wt      LL
## ASRS screen + PGS 5 818.90       0.00   0.87   0.87 -404.42
## ASRS screen       4 822.68       3.78   0.13   1.00 -407.32
```

Now we calculate the weighted logistic R^2 of the different models according to Lee et al. 2012, and the incremental R^2. The following code is the source for Table 4. We first require a weighting factor, which we will calculate using the population prevalence from Song et al. 2021 and our sample prevalence:

```r
summary(ADHD_PGS$Grtilh)
```

```
##   1   2
## 576 530
```

```r
P = 576/1106
K = 0.0258

wv = (1-P)*K/abs(P*(1-K)
                 )

y <- ADHD_PGS$Grtilh

vr = runif(1106,
           0,
           1)
```

First WURS and PGS model:

```r
vsel_WURS = Model_WURS$linear.predictors[y == 2 | vr < wv]

R2_Model_WURS = var(vsel_WURS,
                            na.rm = TRUE)/(var(vsel_WURS,
                            na.rm = TRUE) + pi^(2/3)
                          )
R2_Model_WURS
```

```
## [1] 0.7732602
```

```r
vsel_WURS_PGS = Model_WURS_PGS$linear.predictors[y == 2 |
                                      vr < wv]

R2_Model_WURS_PGS = var(vsel_WURS_PGS,
                            na.rm = TRUE)/(var(vsel_WURS_PGS,
                            na.rm = TRUE) + pi^(2/3)
                              )
R2_Model_WURS_PGS
```

```
## [1] 0.7793635
```

```
R2_PGS_WURS = R2_Model_WURS_PGS - R2_Model_WURS
R2_PGS_WURS
```

```
## [1] 0.006103273
```

Now for ASRS and PGS model:

```
vsel_ASRS = Model_ASRS$linear.predictors[y == 2 | vr < wv]

R2_Model_ASRS = var(vsel_ASRS,
                            na.rm = TRUE)/(var(vsel_ASRS,
                            na.rm = TRUE) + pi^(2/3)
                               )
R2_Model_ASRS
```

```
## [1] 0.6110854
```

```
vsel_ASRS_PGS = Model_ASRS_PGS$linear.predictors[y == 2 | vr < wv]

R2_Model_ASRS_PGS = var(vsel_ASRS_PGS,
                            na.rm = TRUE)/(var(vsel_ASRS_PGS,
                            na.rm = TRUE) + pi^(2/3)
                                       )
R2_Model_ASRS_PGS
```

```
## [1] 0.6168557
```

```
R2_PGS_ASRS = R2_Model_ASRS_PGS - R2_Model_ASRS
R2_PGS_ASRS
```

```
## [1] 0.005770285
```

Now for full model:

```
vsel_WURS_ASRS = Model_WURS_ASRS$linear.predictors[y == 2 | vr < wv]

R2_Model_WURS_ASRS = var(vsel_WURS_ASRS,
                        na.rm = TRUE)/(var(vsel_WURS_ASRS,
                                           na.rm = TRUE) + pi^(2/3)
                                       )
R2_Model_WURS_ASRS
```

```
## [1] 0.8083782
```

```
vsel_WURS_ASRS_PGS = Model_WURS_ASRS_PGS$linear.predictors[y == 2 | vr < wv]

R2_Model_WURS_ASRS_PGS = var(vsel_WURS_ASRS_PGS,
                            na.rm = TRUE)/(var(vsel_WURS_ASRS_PGS,
                                               na.rm = TRUE) + pi^(2/3)
                                           )
R2_Model_WURS_ASRS_PGS
```

```
## [1] 0.8140246
```

```
R2_PGS_WURS_ASRS = R2_Model_WURS_ASRS_PGS - R2_Model_WURS_ASRS
R2_PGS_WURS_ASRS
```

```
## [1] 0.005646386
```

Now for the base model:

```
vsel_PGS = Model_PGS$linear.predictors[y == 2 | vr < wv]

R2_Model_PGS = var(vsel_PGS,
                              na.rm = TRUE)/(var(vsel_PGS,
                              na.rm = TRUE) + pi^(2/3)
                               )
R2_Model_PGS
```

```
## [1] 0.1760275
```

```
vsel_Base = Model_Base$linear.predictors[y == 2 | vr < wv]

R2_Model_Base = var(vsel_Base,
                              na.rm = TRUE)/(var(vsel_Base,
                              na.rm = TRUE) + pi^(2/3)
                                 )
R2_Model_Base
```

```
## [1] 0.1608451
```

```
R2_PGS = R2_Model_PGS - R2_Model_Base
R2_PGS
```

```
## [1] 0.0151824
```

Now for ASRS-screener and PGS model. This is the code for Table S11:

```
vsel_ASRS_screen = Model_ASRS_screen$linear.predictors[y == 2 | vr < wv]

R2_Model_ASRS_screen = var(vsel_ASRS_screen,
                              na.rm = TRUE)/(var(vsel_ASRS_screen,
                              na.rm = TRUE) + pi^(2/3)
                                        )
R2_Model_ASRS_screen
```

```
## [1] 0.5843615
```

```
vsel_ASRS_PGS = Model_ASRS_screen_PGS$linear.predictors[y == 2 | vr < wv]

R2_Model_ASRS_screen_PGS = var(vsel_ASRS_PGS,
                              na.rm = TRUE)/(var(vsel_ASRS_PGS,
                              na.rm = TRUE) + pi^(2/3)
                                                )
R2_Model_ASRS_screen_PGS
```

```
## [1] 0.5909802
```

```
R2_PGS_ASRS_screen = R2_Model_ASRS_screen_PGS - R2_Model_ASRS_screen
R2_PGS_ASRS_screen
```

```
## [1] 0.006618725
```

Logistic Regression with family history as a covariate

Performing the same analysis as described above, with family history as a covariate. WURS-only model:

```
Model_WURS <- glm(Grtilh ~ WURS_tot + Kjonn + Alder + FamADHD,
                  family = binomial(logit),
```

```r
                  data = ADHD_PGS)

summary(Model_WURS)
```

```
##
## Call:
## glm(formula = Grtilh ~ WURS_tot + Kjonn + Alder + FamADHD, family = binomial(logit),
##     data = ADHD_PGS)
##
## Coefficients:
##              Estimate Std. Error z value Pr(>|z|)
## (Intercept)  5.850792   0.601098   9.734  < 2e-16 ***
## WURS_tot    -0.125812   0.008176 -15.388  < 2e-16 ***
## Kjonn2      -0.743550   0.241951  -3.073  0.00212 **
## Alder       -0.062509   0.014252  -4.386 1.15e-05 ***
## FamADHD.L   -1.587561   0.260546  -6.093 1.11e-09 ***
## ---
## Signif. codes:  0 '***' 0.001 '**' 0.01 '*' 0.05 '.' 0.1 ' ' 1
##
## (Dispersion parameter for binomial family taken to be 1)
##
##     Null deviance: 1413.98  on 1019  degrees of freedom
## Residual deviance:  475.92  on 1015  degrees of freedom
##   (86 observations deleted due to missingness)
## AIC: 485.92
##
## Number of Fisher Scoring iterations: 6
```

```r
tidy(Model_WURS)
```

```
## # A tibble: 5 x 5
##   term        estimate std.error statistic  p.value
##   <chr>          <dbl>     <dbl>     <dbl>    <dbl>
## 1 (Intercept)   5.85     0.601        9.73 2.17e-22
## 2 WURS_tot     -0.126    0.00818    -15.4  1.97e-53
## 3 Kjonn2       -0.744    0.242       -3.07 2.12e- 3
## 4 Alder        -0.0625   0.0143      -4.39 1.15e- 5
## 5 FamADHD.L    -1.59     0.261       -6.09 1.11e- 9
```

WURS and PGS model:

```r
Model_WURS_PGS <- glm(Grtilh ~ WURS_tot + Z_res_SCORE + Kjonn + Alder + FamADHD,
                      family = binomial(logit),
                      data = ADHD_PGS)

tidy(Model_WURS_PGS)
```

```
## # A tibble: 6 x 5
##   term        estimate std.error statistic  p.value
##   <chr>          <dbl>     <dbl>     <dbl>    <dbl>
## 1 (Intercept)   5.93     0.608        9.76 1.71e-22
## 2 WURS_tot     -0.126    0.00823    -15.3  6.73e-53
## 3 Z_res_SCORE  -0.254    0.120       -2.11 3.49e- 2
## 4 Kjonn2       -0.747    0.243       -3.07 2.12e- 3
## 5 Alder        -0.0638   0.0144      -4.42 9.85e- 6
## 6 FamADHD.L    -1.53     0.261       -5.87 4.30e- 9
```

ASRS-only model:

```
Model_ASRS <- glm(Grtilh ~ ASRS_tot + Kjonn + Alder + FamADHD,
                  family = binomial(logit),
                  data = ADHD_PGS)

tidy(Model_ASRS)
```

```
## # A tibble: 5 x 5
##   term        estimate std.error statistic  p.value
##   <chr>          <dbl>     <dbl>     <dbl>    <dbl>
## 1 (Intercept)   5.62     0.519       10.8  2.62e-27
## 2 ASRS_tot     -0.140    0.00886    -15.8  3.74e-56
## 3 Kjonn2       -0.980    0.200       -4.89 9.89e- 7
## 4 Alder        -0.0497   0.0120      -4.15 3.33e- 5
## 5 FamADHD.L    -1.80     0.229       -7.88 3.16e-15
```

ASRS and PGS model:

```
Model_ASRS_PGS <- glm(Grtilh ~ ASRS_tot + Z_res_SCORE + Kjonn + Alder + FamADHD,
                      family = binomial(logit),
                      data = ADHD_PGS)

tidy(Model_ASRS_PGS)
```

```
## # A tibble: 6 x 5
##   term        estimate std.error statistic  p.value
##   <chr>          <dbl>     <dbl>     <dbl>    <dbl>
## 1 (Intercept)   5.64     0.521       10.8  2.75e-27
## 2 ASRS_tot     -0.140    0.00889    -15.7  1.33e-55
## 3 Z_res_SCORE  -0.156    0.0997      -1.57 1.17e- 1
## 4 Kjonn2       -0.992    0.201       -4.94 7.87e- 7
## 5 Alder        -0.0503   0.0121      -4.17 2.99e- 5
## 6 FamADHD.L    -1.79     0.229       -7.82 5.49e-15
```

ASRS-screen model:

```
Model_ASRS_screen <- glm(Grtilh ~ ASRSscreen + Kjonn + Alder + FamADHD,
                         family = binomial(logit),
                         data = ADHD_PGS)

tidy(Model_ASRS_screen)
```

```
## # A tibble: 5 x 5
##   term        estimate std.error statistic  p.value
##   <chr>          <dbl>     <dbl>     <dbl>    <dbl>
## 1 (Intercept)   5.44      0.498      10.9  8.89e-28
## 2 ASRSscreen   -0.362     0.0233    -15.5  1.62e-54
## 3 Kjonn2       -0.920     0.192      -4.79 1.66e- 6
## 4 Alder        -0.0584    0.0116     -5.06 4.26e- 7
## 5 FamADHD.L    -1.78      0.223      -7.98 1.41e-15
```

ASRS-screen and PGS model:

```
Model_ASRS_screen_PGS <- glm(Grtilh ~ ASRSscreen + Z_res_SCORE + Kjonn + Alder +
                               FamADHD,
                             family = binomial(logit),
                             data = ADHD_PGS)
```

```r
tidy(Model_ASRS_screen_PGS)
```

```
## # A tibble: 6 x 5
##   term        estimate std.error statistic  p.value
##   <chr>          <dbl>     <dbl>     <dbl>    <dbl>
## 1 (Intercept)   5.48      0.502      10.9  8.58e-28
## 2 ASRSscreen   -0.361     0.0234    -15.4  8.01e-54
## 3 Z_res_SCORE  -0.166     0.0958     -1.73 8.33e- 2
## 4 Kjonn2       -0.934     0.193      -4.85 1.25e- 6
## 5 Alder        -0.0595    0.0116     -5.11 3.25e- 7
## 6 FamADHD.L    -1.75      0.222      -7.88 3.24e-15
```

WURS and ASRS model:

```r
Model_WURS_ASRS <- glm(Grtilh ~ WURS_tot + ASRS_tot + Kjonn + Alder + FamADHD,
                       family = binomial(logit),
                       data = ADHD_PGS)
tidy(Model_WURS_ASRS)
```

```
## # A tibble: 6 x 5
##   term        estimate std.error statistic  p.value
##   <chr>          <dbl>     <dbl>     <dbl>    <dbl>
## 1 (Intercept)   6.94     0.695        9.99 1.70e-23
## 2 WURS_tot     -0.0996   0.00873    -11.4  3.68e-30
## 3 ASRS_tot     -0.0679   0.0114      -5.96 2.48e- 9
## 4 Kjonn2       -0.900    0.259       -3.47 5.17e- 4
## 5 Alder        -0.0490   0.0152      -3.22 1.26e- 3
## 6 FamADHD.L    -1.50     0.275       -5.44 5.22e- 8
```

WURS, ASRS and PGS model:

```r
Model_WURS_ASRS_PGS <- glm(Grtilh ~ WURS_tot + ASRS_tot + Z_res_SCORE + Kjonn
                           + Alder + FamADHD,
                           family = binomial(logit),
                           data = ADHD_PGS)
tidy(Model_WURS_ASRS_PGS)
```

```
## # A tibble: 7 x 5
##   term        estimate std.error statistic  p.value
##   <chr>          <dbl>     <dbl>     <dbl>    <dbl>
## 1 (Intercept)   7.03     0.706        9.96 2.24e-23
## 2 WURS_tot     -0.101    0.00880    -11.4  2.57e-30
## 3 ASRS_tot     -0.0675   0.0114      -5.92 3.13e- 9
## 4 Z_res_SCORE  -0.252    0.127       -1.99 4.68e- 2
## 5 Kjonn2       -0.907    0.260       -3.49 4.89e- 4
## 6 Alder        -0.0504   0.0154      -3.28 1.06e- 3
## 7 FamADHD.L    -1.44     0.275       -5.26 1.45e- 7
```

PGS model:

```r
Model_PGS <- glm(Grtilh ~ Z_res_SCORE + Kjonn + Alder + FamADHD,
                 family = binomial(logit),
                 data = ADHD_PGS)

tidy(Model_PGS)
```

```
## # A tibble: 5 x 5
```

```
##   term        estimate std.error statistic  p.value
##   <chr>          <dbl>     <dbl>     <dbl>    <dbl>
## 1 (Intercept)   1.49     0.302        4.92 8.74e- 7
## 2 Z_res_SCORE  -0.250    0.0728      -3.43 5.97e- 4
## 3 Kjonn2       -0.671    0.143       -4.71 2.53e- 6
## 4 Alder        -0.0707   0.00867     -8.16 3.49e-16
## 5 FamADHD.L    -1.89     0.182      -10.4  2.11e-25
```

Base model:

```
Model_Base <- glm(Grtilh ~ Kjonn + Alder + FamADHD,
                  family = binomial(logit),
                  data = ADHD_PGS)

tidy(Model_Base)
```

```
## # A tibble: 4 x 5
##   term        estimate std.error statistic  p.value
##   <chr>          <dbl>     <dbl>     <dbl>    <dbl>
## 1 (Intercept)   1.46     0.300        4.88 1.05e- 6
## 2 Kjonn2       -0.673    0.142       -4.75 2.07e- 6
## 3 Alder        -0.0701   0.00858     -8.17 3.20e-16
## 4 FamADHD.L    -1.91     0.181      -10.5  7.60e-26
```

PGS only model:

```
Model_PGS_only <- glm(Grtilh ~ Z_res_SCORE,
                      family = binomial(logit),
                      data = ADHD_PGS)

tidy(Model_PGS_only)
```

```
## # A tibble: 2 x 5
##   term        estimate std.error statistic   p.value
##   <chr>          <dbl>     <dbl>     <dbl>     <dbl>
## 1 (Intercept)  -0.0859    0.0607     -1.42 0.157
## 2 Z_res_SCORE  -0.251     0.0620     -4.05 0.0000503
```

Likelihood ratio tests. This is the source for Table S4.

```
lrtest(Model_Base,
       Model_PGS)
```

```
## Likelihood ratio test
##
## Model 1: Grtilh ~ Kjonn + Alder + FamADHD
## Model 2: Grtilh ~ Z_res_SCORE + Kjonn + Alder + FamADHD
##   #Df  LogLik Df  Chisq Pr(>Chisq)
## 1   4 -598.37
## 2   5 -592.35  1 12.036  0.0005217 ***
## ---
## Signif. codes:  0 '***' 0.001 '**' 0.01 '*' 0.05 '.' 0.1 ' ' 1
```

```
lrtest(Model_WURS_ASRS,
       Model_WURS_ASRS_PGS)
```

```
## Likelihood ratio test
##
```

```
## Model 1: Grtilh ~ WURS_tot + ASRS_tot + Kjonn + Alder + FamADHD
## Model 2: Grtilh ~ WURS_tot + ASRS_tot + Z_res_SCORE + Kjonn + Alder +
##     FamADHD
##   #Df  LogLik Df  Chisq Pr(>Chisq)
## 1   6 -216.48
## 2   7 -214.50  1 3.9687    0.04635 *
## ---
## Signif. codes:  0 '***' 0.001 '**' 0.01 '*' 0.05 '.' 0.1 ' ' 1
```

```
lrtest(Model_WURS,
       Model_WURS_PGS)
```

```
## Likelihood ratio test
##
## Model 1: Grtilh ~ WURS_tot + Kjonn + Alder + FamADHD
## Model 2: Grtilh ~ WURS_tot + Z_res_SCORE + Kjonn + Alder + FamADHD
##   #Df  LogLik Df  Chisq Pr(>Chisq)
## 1   5 -237.96
## 2   6 -235.72  1 4.4747     0.0344 *
## ---
## Signif. codes:  0 '***' 0.001 '**' 0.01 '*' 0.05 '.' 0.1 ' ' 1
```

```
lrtest(Model_ASRS,
       Model_ASRS_PGS)
```

```
## Likelihood ratio test
##
## Model 1: Grtilh ~ ASRS_tot + Kjonn + Alder + FamADHD
## Model 2: Grtilh ~ ASRS_tot + Z_res_SCORE + Kjonn + Alder + FamADHD
##   #Df  LogLik Df  Chisq Pr(>Chisq)
## 1   5 -342.96
## 2   6 -341.73  1 2.4578     0.1169
```

Now we calculate the AICc of the different models. This is the source for Table S10.

```
models <- list(Model_WURS, Model_WURS_PGS, Model_ASRS, Model_ASRS_PGS,
               Model_WURS_ASRS, Model_WURS_ASRS_PGS, Model_PGS, Model_Base,
               Model_ASRS_screen, Model_ASRS_screen_PGS)

model.names <- c("WURS", "WURS+PGS",  "ASRS", "ASRS+PGS", "ASRS+WURS",
                 "ASRS+WURS+PGS", "PGS", "Base",
                 "ASRS screen", "ASRS screen + PGS")

model_aic <- aictab(cand.set = models,
                    modnames = model.names)

model_aic
```

```
##
## Model selection based on AICc:
##
##                     K    AICc Delta_AICc AICcWt Cum.Wt      LL
## ASRS+WURS+PGS       7  443.10       0.00   0.73   0.73 -214.49
## ASRS+WURS           6  445.04       1.94   0.27   1.00 -216.48
## WURS+PGS            6  483.53      40.43   0.00   1.00 -235.72
## WURS                5  485.98      42.88   0.00   1.00 -237.96
## ASRS+PGS            6  695.54     252.44   0.00   1.00 -341.73
```

```
## ASRS              5  695.98     252.88   0.00    1.00 -342.96
## ASRS screen + PGS 6  738.89     295.78   0.00    1.00 -363.40
## ASRS screen       5  739.87     296.77   0.00    1.00 -364.91
## PGS               5 1194.76     751.66   0.00    1.00 -592.35
## Base              4 1204.78     761.67   0.00    1.00 -598.37
```

Now we calculate the weighted R^2 on the logistic liability model of the different models and the incremental Lee R^2. The following code is the source for Table S12.

First WURS and PGS model:

```
vsel_WURS = Model_WURS$linear.predictors[y == 2 | vr < wv]

R2_Model_WURS = var(vsel_WURS,
                            na.rm = TRUE)/(var(vsel_WURS,
                            na.rm = TRUE) + pi^(2/3)
                           )
R2_Model_WURS
```

```
## [1] 0.798729
```

```
vsel_WURS_PGS = Model_WURS_PGS$linear.predictors[y == 2 |
                                      vr < wv]

R2_Model_WURS_PGS = var(vsel_WURS_PGS,
                            na.rm = TRUE)/(var(vsel_WURS_PGS,
                            na.rm = TRUE) + pi^(2/3)
                               )
R2_Model_WURS_PGS
```

```
## [1] 0.800771
```

```
R2_PGS_WURS = R2_Model_WURS_PGS - R2_Model_WURS
R2_PGS_WURS
```

```
## [1] 0.002042008
```

Now for ASRS and PGS model:

```
vsel_ASRS = Model_ASRS$linear.predictors[y == 2 | vr < wv]

R2_Model_ASRS = var(vsel_ASRS,
                            na.rm = TRUE)/(var(vsel_ASRS,
                            na.rm = TRUE) + pi^(2/3)
                                )
R2_Model_ASRS
```

```
## [1] 0.6559318
```

```
vsel_ASRS_PGS = Model_ASRS_PGS$linear.predictors[y == 2 | vr < wv]

R2_Model_ASRS_PGS = var(vsel_ASRS_PGS,
                            na.rm = TRUE)/(var(vsel_ASRS_PGS,
                            na.rm = TRUE) + pi^(2/3)
                                        )
R2_Model_ASRS_PGS
```

```
## [1] 0.6592011
```

```r
R2_PGS_ASRS = R2_Model_ASRS_PGS - R2_Model_ASRS
R2_PGS_ASRS
```

```
## [1] 0.003269307
```

Now for full model:

```r
vsel_WURS_ASRS = Model_WURS_ASRS$linear.predictors[y == 2 | vr < wv]

R2_Model_WURS_ASRS = var(vsel_WURS_ASRS,
                        na.rm = TRUE)/(var(vsel_WURS_ASRS,
                                           na.rm = TRUE) + pi^(2/3)
                                       )
R2_Model_WURS_ASRS
```

```
## [1] 0.8213912
```

```r
vsel_WURS_ASRS_PGS = Model_WURS_ASRS_PGS$linear.predictors[y == 2 | vr < wv]

R2_Model_WURS_ASRS_PGS = var(vsel_WURS_ASRS_PGS,
                            na.rm = TRUE)/(var(vsel_WURS_ASRS_PGS,
                                               na.rm = TRUE) + pi^(2/3)
                                           )
R2_Model_WURS_ASRS_PGS
```

```
## [1] 0.8239175
```

```r
R2_PGS_WURS_ASRS = R2_Model_WURS_ASRS_PGS - R2_Model_WURS_ASRS
R2_PGS_WURS_ASRS
```

```
## [1] 0.002526268
```

Now for the base model:

```r
vsel_PGS = Model_PGS$linear.predictors[y == 2 | vr < wv]

R2_Model_PGS = var(vsel_PGS,
                            na.rm = TRUE)/(var(vsel_PGS,
                            na.rm = TRUE) + pi^(2/3)
                             )
R2_Model_PGS
```

```
## [1] 0.2945678
```

```r
vsel_Base = Model_Base$linear.predictors[y == 2 | vr < wv]

R2_Model_Base = var(vsel_Base,
                            na.rm = TRUE)/(var(vsel_Base,
                            na.rm = TRUE) + pi^(2/3)
                               )
R2_Model_Base
```

```
## [1] 0.2806159
```

```r
R2_PGS = R2_Model_PGS - R2_Model_Base
R2_PGS
```

```
## [1] 0.01395193
```

Now for ASRS-screener and PGS model:

```
vsel_ASRS_screen = Model_ASRS_screen$linear.predictors[y == 2 | vr < wv]

R2_Model_ASRS_screen = var(vsel_ASRS_screen,
                           na.rm = TRUE)/(var(vsel_ASRS_screen,
                           na.rm = TRUE) + pi^(2/3)
                                      )
R2_Model_ASRS_screen
```

```
## [1] 0.628524
```

```
vsel_ASRS_PGS = Model_ASRS_screen_PGS$linear.predictors[y == 2 | vr < wv]

R2_Model_ASRS_screen_PGS = var(vsel_ASRS_PGS,
                           na.rm = TRUE)/(var(vsel_ASRS_PGS,
                           na.rm = TRUE) + pi^(2/3)
                                              )
R2_Model_ASRS_screen_PGS
```

```
## [1] 0.6312183
```

```
R2_PGS_ASRS_screen = R2_Model_ASRS_screen_PGS - R2_Model_ASRS_screen
R2_PGS_ASRS_screen
```

```
## [1] 0.00269433
```

Sensitivity Analysis

Finally, we analyze whether the ADHD PGS is significantly associated with WURS-score, ASRS-score and family history of ADHD when controlling for age, sex and the first 5 PCs. This is the source for Table S13:

```
WURS_PGS <- lm(WURS_tot ~ Z_res_SCORE + Kjonn + Alder,
                  data = ADHD_PGS)
tidy(WURS_PGS)
```

```
## # A tibble: 4 x 5
##   term        estimate std.error statistic  p.value
##   <chr>          <dbl>     <dbl>     <dbl>    <dbl>
## 1 (Intercept)   14.8      2.83        5.21 2.30e- 7
## 2 Z_res_SCORE    2.85     0.769       3.70 2.25e- 4
## 3 Kjonn2         4.67     1.55        3.01 2.72e- 3
## 4 Alder          0.645    0.0851      7.57 8.36e-14
```

```
ASRS_PGS <- lm(ASRS_tot ~ Z_res_SCORE + Kjonn + Alder,
                  data = ADHD_PGS)
tidy(ASRS_PGS)
```

```
## # A tibble: 4 x 5
##   term        estimate std.error statistic  p.value
##   <chr>          <dbl>     <dbl>     <dbl>    <dbl>
## 1 (Intercept)  19.7       1.70     11.6    2.24e-29
## 2 Z_res_SCORE   1.90      0.467     4.07   4.98e- 5
## 3 Kjonn2       -0.0239    0.936    -0.0256 9.80e- 1
## 4 Alder         0.461     0.0505    9.12   3.54e-19
```

```
ASRSscreen_PGS <- lm(ASRSscreen ~ Z_res_SCORE + Kjonn + Alder,
                  data = ADHD_PGS)
```

```r
tidy(ASRSscreen_PGS)
```

```
## # A tibble: 4 x 5
##   term        estimate std.error statistic  p.value
##   <chr>          <dbl>     <dbl>     <dbl>    <dbl>
## 1 (Intercept)    7.00     0.634     11.0   6.88e-27
## 2 Z_res_SCORE    0.674    0.175      3.85  1.26e- 4
## 3 Kjonn2         0.104    0.352      0.295 7.68e- 1
## 4 Alder          0.157    0.0189     8.30  3.20e-16
```

```r
Fam_ADHD_PGS <- glm(FamADHD ~ Z_res_SCORE + Kjonn + Alder,
                      family = binomial(logit),
                      data = ADHD_PGS)

tidy(Fam_ADHD_PGS)
```

```
## # A tibble: 4 x 5
##   term        estimate std.error statistic  p.value
##   <chr>          <dbl>     <dbl>     <dbl>    <dbl>
## 1 (Intercept)  -3.55     0.295      -12.1  1.77e-33
## 2 Z_res_SCORE   0.160    0.0771       2.07 3.85e- 2
## 3 Kjonn2       -0.372    0.158       -2.36 1.82e- 2
## 4 Alder         0.0734   0.00819      8.97 2.96e-19
```
