# Supplementary material for: Using polygenic scores in combination with symptom rating scales to identify attention-deficit/hyperactivity disorder
Source: BMC Psychiatry. 2024 Jun 27;24:471. doi: 10.1186/s12888-024-05925-7 (PMC11210094; doi:10.1186/s12888-024-05925-7)
Supplement: Supplementary file 2 — Supplementary Material 2 [file 12888_2024_5925_MOESM2_ESM.docx]

# Additional files

Additional File 1

File format: .pdf

Title of data: Additional File 1

Additional File 1 contains the R-code employed in the production of this article’s results in an R Markdown-document.

Additional File 2

File format: .docx

Title of data: Supplementary Tables

Additional File 2 contains supplementary tables showing the results of sub-analyses including the ASRS-screener and self-reported family history of ADHD as predictors of ADHD diagnosis.
