# Supplementary material for: Using polygenic scores in combination with symptom rating scales to identify attention-deficit/hyperactivity disorder
Source: BMC Psychiatry. 2024 Jun 27;24:471. doi: 10.1186/s12888-024-05925-7 (PMC11210094; doi:10.1186/s12888-024-05925-7)
Supplement: Supplementary file 3 — Supplementary Material 3 [file 12888_2024_5925_MOESM3_ESM.docx]

# Supplementary Tables

**Table S1:** **Comparing rating scales and PGS between males and females**

| **Table S1** | Male | Female | Degrees of freedom^a^ | p^a^ |
| --- | --- | --- | --- | --- |
| **Mean ASRS score (SD)** | 34.14 (15.7) | 34.1 (16.1) | 1018.3 | 0.9998 |
| **Mean WURS score (SD)** | 39.5 (25.6) | 34.7 (25.4) | 964.72 | 0.0029 |
| **Mean PGS (SD)** | -0.009 (1.03) | -0.003 (0.97) | 1005.8 | 0.9227 |

*Table S1. a. Degrees of freedom and p refer to the degrees of freedom and results of t-tests comparing the groups. ASRS: adult ADHD self-report scale, PGS: polygenic score, WURS: Wender-Utah rating scale.*

**Table S2:** **Comparing rating scales and PGS between individuals with and without family history of ADHD**

| **Table S2** | Family history | No family history | Degrees of freedom^a^ | *p*^a^ |
| --- | --- | --- | --- | --- |
| **Mean ASRS score (SD)** | 43.7 (13.4) | 31.4 (15.5) | 421.73 | <0.0001 |
| **Mean WURS score (SD)** | 53.2(20.6) | 32.4 (24.9) | 398.45 | <0.0001 |
| **Mean PGS (SD)** | 0.014 (1.01) | -0.042 (0.99) | 374.97 | 0.0158 |

*Table S2. a. Degrees of freedom and p refer to the degrees of freedom and results of t-tests comparing the groups. ASRS: adult ADHD self-report scale, PGS: polygenic score, WURS: Wender-Utah rating scale.*

**Table S3:** **Results of LR tests comparing models including ASRS screener with and without ADHD PGS**

| **Table S3** | Delta chi-square (1) | *p* |
| --- | --- | --- |
| ASRS-screener + PGS vs. ASRS-screener | 5.8 | 0.016 |

*Table S3. ASRS: adult ADHD self-report scale, PGS: polygenic score.*

**Table S4:** **Results of LR tests comparing models including ADHD family history with and without ADHD PGS**

| **Table S4** | Delta chi-square (1) | *p* |
| --- | --- | --- |
| PGS vs. base model^a^ | 12.0 | 0.0005 |
| ASRS + PGS vs. ASRS | 2.46 | 0.1169 |
| ASRS-screener + PGS vs. ASRS-screener | 3.01 | 0.0829 |
| WURS + PGS vs. WURS | 4.47 | 0.0344 |
| ASRS + WURS + PGS vs. ASRS + WURS | 3.97 | 0.0464 |

*Table S4. a. The base model includes sex, age and family history. ASRS: adult ADHD self-report scale, PGS: polygenic score, WURS: Wender-Utah rating scale.*

**Table S5: Comparison of hierarchical full model by AICc**

| **Table S5** | K | AICc | ΔAICc |
| --- | --- | --- | --- |
| ASRS + WURS + PGS | 6 | 474.96 | - |
| ASRS + WURS | 5 | 479.52 | 4.56 |

*Table S5.* *K is the number of estimated parameters in the model. ΔAICc is the difference in AICc of the best-fit model and the respective models. AICc: Akaike Information Criterion corrected, ASRS: adult ADHD self-report scale, PGS: polygenic score, WURS: Wender-Utah rating scale.*

**Table S6: Comparison of hierarchical WURS-models by AICc**

| **Table S6** | K | AICc | ΔAICc |
| --- | --- | --- | --- |
| WURS+ PGS | 5 | 523.69 | - |
| WURS | 4 | 529.62 | 5.93 |

*Table S6.* *K is the number of estimated parameters in the model. ΔAICc is the difference in AICc of the best-fit model and the respective models. AICc: Akaike Information Criterion corrected, PGS: polygenic score, WURS: Wender-Utah rating scale.*

**Table S7: Comparison of hierarchical ASRS-models by AICc**

| **Table S7** | K | AICc | ΔAICc |
| --- | --- | --- | --- |
| ASRS + PGS | 5 | 774.79 | - |
| ASRS | 4 | 777.07 | 2.28 |

*Table S7.* *K is the number of estimated parameters in the model. ΔAICc is the difference in AICc of the best-fit model and the respective models. AICc: Akaike Information Criterion corrected, ASRS: adult ADHD self-report scale, PGS: polygenic score.*

**Table S8: Comparison of hierarchical base models by AICc**

| **Table S8** | K | AICc | ΔAICc |
| --- | --- | --- | --- |
| PGS | 4 | 1374.57 | - |
| Base model^a^ | 3 | 1388.27 | 13.7 |

*Table S8.* a: *The base model includes sex and age. K is the number of estimated parameters in the model. ΔAICc is the difference in AICc of the best-fit model and the respective models. AICc: Akaike Information Criterion corrected, PGS: polygenic score.*

**Table S9: Comparison of logistic regression models including ASRS screener with and without ADHD PGS by AICc**

| **Table S9** | K | AICc | ΔAICc |
| --- | --- | --- | --- |
| ASRS screener + PGS | 5 | 818.90 | - |
| ASRS screener | 4 | 822.68 | 3.78 |

*Table S9.* *K is the number of estimated parameters in the model. ΔAICc is the difference in AICc of the best-fit model and the respective models. AICc: Akaike Information Criterion corrected for small sample sizes, ASRS: adult ADHD self-report scale, PGS: polygenic score.*

**Table S10: Comparison of logistic regression models including ADHD family history by AICc**

| **Table S10** | K | AICc | ΔAICc |
| --- | --- | --- | --- |
| ASRS + WURS + PGS | 7 | 443.10 | - |
| ASRS + WURS | 6 | 445.04 | 1.94 |
| WURS + PGS | 6 | 483.53 | 40.43 |
| WURS | 5 | 485.98 | 42.88 |
| ASRS + PGS | 6 | 695.54 | 252.44 |
| ASRS | 5 | 695.98 | 252.88 |
| ASRS screener + PGS | 6 | 738.89 | 295.78 |
| ASRS screener | 5 | 739.87 | 296.77 |
| PGS | 5 | 1194.76 | 751.66 |
| Base model^a^ | 4 | 1204.78 | 761.67 |

*Table S10.* *a. The base model includes sex, age, and family history. K is the number of estimated parameters in the model. ΔAICc is the difference in AICc of the best-fit model and the respective models. AICc: Akaike Information Criterion corrected for small sample sizes, ASRS: adult ADHD self-report scale, PGS: polygenic score, WURS: Wender-Utah rating scale*

**Table S11. Lee R^2^ by hierarchical logistic regression models including ASRS screener with and without ADHD PGS**

| **Table S11** | Lee R^2^ | Incremental R^2^ by ADHD PGS |
| --- | --- | --- |
| ASRS screener + PGS | 59.098% | 0.662pp |
| ASRS screener | 58.436% | --- |

*Table S11.* *ASRS: adult ADHD self-report scale, PGS: polygenic score, pp: percentage points.*

**Table S12. Lee R^2^ by different logistic regression models including ADHD family history**

| **Table S12** | Lee R^2^ | Incremental R^2^ by ADHD PGS |
| --- | --- | --- |
| ASRS + WURS + PGS | 82.392% | 0.253pp |
| ASRS + WURS | 82.139% | --- |
| WURS + PGS | 80.077% | 0.204pp |
| WURS | 79.872% | --- |
| ASRS + PGS | 65.920% | 0.327pp |
| ASRS | 65.593% | --- |
| ASRS screener + PGS | 63.122% | 0.269pp |
| ASRS screener | 62.852% | --- |
| PGS | 29.457% | 1.395pp |
| Base model^a^ | 28.062% | --- |

*Table S12.* *a. The base model includes sex, age, and family history. ASRS: Adult ADHD Self-Report Scale, PGS: polygenic score, pp: percentage points, WURS: Wender-Utah Rating Scale.*

**Table S13. Results of sensitivity analyses**

| **Table S13** | β ADHD PGS (SE) | ADHD PGS *p*-value |
| --- | --- | --- |
| Linear regression: WURS ~ PGS + Sex + Age | 2.85 (0.769) | <0.001 |
| Linear regression: ASRS ~ PGS + Sex + Age | 1.90 (0.467) | <0.001 |
| Logistic regression:  Family history of ADHD ~ PGS + Sex + Age | 0.16 (0.077) | 0.039 |
| Linear regression: ASRS screener ~ PGS + Sex + Age | 0.67 (0.18) | <0.001 |

*Table S13. ASRS: Adult ADHD Self-Report Scale, PGS: polygenic score, WURS: Wender-Utah Rating Scale.*
